# Histone deficiency and hypoacetylation in the aging retinal pigment epithelium

**DOI:** 10.1111/acel.14108

**Published:** 2024-02-26

**Authors:** Sushil K. Dubey, Rashmi Dubey, Subhash C. Prajapati, Kyungsik Jung, Kabhilan Mohan, Xinan Liu, Jacob Roney, Wenjian Tian, Jennifer Abney, Michelle M. Giarmarco, Alvaro G. Hernandez, Jinze Liu, Mark E. Kleinman

**Affiliations:** ^1^ Department of Surgery East Tennessee State University Johnson City Tennessee USA; ^2^ Department of Biochemistry and Molecular Genetics University of Virginia Charlottesville Virginia USA; ^3^ Department of Computer Science University of Kentucky Lexington Kentucky USA; ^4^ Department of Ophthalmology and Visual Sciences University of Kentucky Lexington Kentucky USA; ^5^ Department of Ophthalmology University of Washington Seattle Washington USA; ^6^ Roy J. Carver Biotechnology Center University of Illinois at Urbana‐Champaign Urbana Illinois USA; ^7^ Department of Biostatistics Virginia Commonwealth University Richmond Virginia USA

**Keywords:** aging, epigenetics, HINFP, histone acetylation, histones, replicative senescence, retinal pigment epithelium

## Abstract

Histones serve as a major carrier of epigenetic information in the form of post‐translational modifications which are vital for controlling gene expression, maintaining cell identity, and ensuring proper cellular function. Loss of histones in the aging genome can drastically impact the epigenetic landscape of the cell leading to altered chromatin structure and changes in gene expression profiles. In this study, we investigated the impact of age‐related changes on histone levels and histone acetylation in the retinal pigment epithelium (RPE) and retina of mice. We observed a global reduction of histones H1, H2A, H2B, H3, and H4 in aged RPE/choroid but not in the neural retina. Transcriptomic analyses revealed significant downregulation of histones in aged RPE/choroid including crucial elements of the histone locus body (HLB) complex involved in histone pre‐mRNA processing. Knockdown of *HINFP*, a key HLB component, in human RPE cells induced histone loss, senescence, and the upregulation of senescence‐associated secretory phenotype (SASP) markers. Replicative senescence and chronological aging in human RPE cells similarly resulted in progressive histone loss and acquisition of the SASP. Immunostaining of human retina sections revealed histone loss in RPE with age. Acetyl‐histone profiling in aged mouse RPE/choroid revealed a specific molecular signature with loss of global acetyl‐histone levels, including H3K14ac, H3K56ac, and H4K16ac marks. These findings strongly demonstrate histone loss as a unique feature of RPE aging and provide critical insights into the potential mechanisms linking histone dynamics, cellular senescence, and aging.

## Graphical abstract

In this study, we demonstrated an age‐related loss of core and linker histones in aged mouse and human donor RPE with correlating results in vitro. Depleting histones in vitro reduced cell proliferation and upregulated SASP markers. Furthermore, a notable reduction in histone acetylation, a classical histone PTM, was observed in aged mice RPE. Thus, the altered histone expression and acetylation status with age may be a critical epigenetic component of RPE cell aging and senescence.

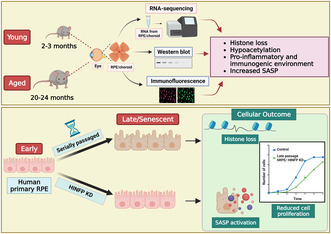

**Abbreviations**
AMD: Age‐related macular degeneration
HLB: Histone Locus Body
PDL: Population doubling level
PTM: Post‐translational modifications
RPE: Retinal pigment epithelium
SASP: Senescence‐associated secretory phenotype
SA‐β‐Gal: Senescence‐associated β‐galactosidase

## 1 INTRODUCTION

The retina is a multilayered light‐sensitive tissue mainly composed of neuronal cells derived from the neuroectoderm. Age‐related retinal diseases such as age‐related macular degeneration (AMD), diabetic retinopathy, and retinitis pigmentosa result from progressive degeneration of the retina (J. B. Lin et al., 2016). These conditions lead to severe visual impairment which can contribute to reduced quality of life and disability in older adults (J. B. Lin et al., 2016; Prem Senthil et al., 2017). The retina is organized into two main components, neural retina and retinal pigment epithelium (RPE). The RPE is a single layer of epithelial cells that plays a crucial role in maintaining retinal homeostasis. It supports the visual cycle metabolism, protects the photoreceptor layer, and removes metabolic waste from the outer retina to the choroid (Bok, 1993). RPE cells are known to be postmitotic with limited proliferative capacity (M. Chen et al., 2016). As RPE cells age, they undergo various changes, including oxidative stress, mitochondrial dysfunction, and increased inflammatory gene expression, leading to the degeneration of RPE and age‐related ocular disorders like AMD (Bonilha, 2008; H. Chen et al., 2008). Elucidating the molecular mechanisms that drive RPE aging is critical to our fundamental scientific understanding of cellular longevity and to developing novel effective therapies for age‐related retinal diseases.

The aging process is driven by changes in genetic and epigenetic information over time (Lopez‐Otin et al., 2013). However, an organism's genome remains relatively stable during its lifetime, and there is a growing interest in research that challenges the notion of genetic aberrations as the primary cause of aging (Yang et al., 2023). In support of this hypothesis are studies that demonstrate normal cellular aging can occur despite fewer mutations, whereas cells with a higher mutation rate do not prematurely age (De Majo et al., 2021; Kaya et al., 2015; Narayanan et al., 1997). Mounting evidence from yeast to humans indicates that loss of epigenetic information, rather than genetic mutations, is a critical aging factor (Liu et al., 2013; Sinclair et al., 1997; Yang et al., 2023). Epigenetic studies in aging models emphasize the loss of heterochromatin and the resulting genome instability as a key contributor to cellular aging and senescence (Tsurumi & Li, 2012). Further research has expanded this perspective with more epigenetic alterations identified in aging cells, including loss of nucleosomes, altered histone modifications and levels, changes in DNA methylation, and noncoding RNA signatures (Pal & Tyler, 2016; Sen et al., 2016). While many studies in aging and age‐related diseases of the retina have focused on microRNA and altered DNA methylation patterns (Berdasco et al., 2017; Porter et al., 2019), there is much less known about histone gene expression profiles and post‐translational modifications (PTMs) in the aging RPE and retina. This knowledge gap exists partly because of the involvement of many genes that encode the different histones and complexity in the synthesis and processing of different histones, regulated at multiple levels by a vast repertoire of transcription and epigenetic factors (Marzluff et al., 2002; Marzluff & Koreski, 2017; Mendiratta et al., 2019). H1, H2A, H2B, H3, and H4 are the five primary types of histones, and multiple copies of genes are organized as clusters in eukaryotic genomes that encode every individual histone. Synthesis of canonical core and linker histone proteins is a highly cell cycle coordinated event tightly regulated by a complex group of transcription factors (Ewen, 2000). These unique non‐polyadenylated mRNAs are processed by an evolutionarily conserved nuclear body known as the histone locus body (HLB) (Nizami et al., 2010). The HLB complex comprises several proteins involved in the pathways of histone biosynthesis and histone RNA transcript processing. The HLB components, HINFP (Histone Nuclear Factor P), NPAT (Nuclear Protein Ataxia‐Telangiectasia), and CASP8AP2 (Caspase 8 Associated Protein 2)/FLASH (FLICE‐associated huge protein) are particularly essential to synthesize abundant quantities of histones during S‐phase of cell cycle and necessary for nucleosome complex assembly (Barcaroli et al., 2006; Ghule et al., 2014; Ye et al., 2003).

In this study, we demonstrated a significant reduction in core and linker histone components H1, H2A, H2B, H3, and H4 along with critical regulatory factors in the HLB, including *Hinfp*, *Npat*, and *Casp8ap2* in the aged mouse RPE/choroid. We also observed downregulation of histone expression in aged human RPE tissues and in vitro aging models utilizing human RPE (hRPE) cells. Through targeted knockdown of *HINFP* in hRPE, we discovered that decreased histone expression leads to the acquisition of a senescence‐associated secretory phenotype (SASP). Our study provides valuable insights into the involvement of histone loss and hypoacetylation in the normal aging process of RPE cells and provides a further understanding of the complex etiology of aging in RPE tissue.

## 2 MATERIALS AND METHODS

A detailed description of the materials and methods (mRNA‐sequencing, analysis of mRNA‐seq data, Western blot analysis, Immunofluorescence, Immunohistochemistry, Quantitative real‐time PCR (qPCR), cell proliferation assay, and SA‐β‐Galactosidase assay.) is available in Appendix S1.

### 2.1 Animals

All mouse experiments were conducted in compliance with the guidelines established by the Association for Research in Vision and Ophthalmology for the Use of Animals in Ophthalmic and Vision Research. Male and female wild‐type C57BL/6J mice aged between 2 and 3 months were included in the young group, while mice aged between 20 and 24 months were assigned to the aged group. The mice were purchased from the Jackson Laboratory and housed under standard conditions (23 ± 1°C, 40%–50% humidity, and ad libitum access to food and water). The research protocol was approved by the Institutional Animal Care and Use Committee of the University of Kentucky and East Tennessee State University.

### 2.2 Mouse retina and RPE collection

For the collection of mouse retina and RPE, the isolated eyes were placed in the cold petri dish under a surgical microscope. With careful precision, the anterior parts, including the cornea, lens, and iris, as well as connective tissues, muscles, and optic nerve, were excised. Radial cuts were made symmetrically in a four‐leaf pattern, allowing for the careful removal of the neural retina from the mouse eyecup. The pigmented RPE/choroid layer was gently scraped from the sclera. RPE/choroid and neural retina tissues were immediately transferred to ice‐cold 1X RIPA buffer (Thermo Scientific 89,900, Rockford, IL) for cell lysate preparation, 1X pre‐lysis buffer (Epigentek OP‐0006, Farmingdale, NY) for histone extract preparation, or RNA lysis buffer (Invitrogen 12183016, Carlsbad, CA) for RNA isolation, based on the specific experimental requirements. The tissues were snap‐frozen and stored in liquid nitrogen to keep them intact if not used immediately.

### 2.3 RNA sequencing

For RNA sequencing analysis, RPE/choroid tissue (two eyes/sample) was collected from young and aged mice (*n* = 3 per group). RNA extraction was performed using TRIzol reagent (Invitrogen 15596026, Carlsbad, CA) according to the manufacturer's instructions. RNA purification, library preparation, sequencing, and analysis procedures are described in the Appendix S1, *Materials and Methods*.

### 2.4 Histone extraction

Histones were extracted from tissues and RPE cell lines using the EpiQuik Total Histone Extraction Kit (Epigentek OP‐0006, Farmingdale, NY) following the manufacturer's instructions (See Appendix S1, *Materials and Methods* for more details).

### 2.5 Human RPE cell culture and transfection

Details regarding Human RPE culture and transfection using siRNA are provided in the Appendix S1, *Materials and Methods*.

### 2.6 In vitro replicative aging in hRPE cells

Preparation of naturally passaged aged hRPE cells was performed as described previously (Matsunaga et al., 1999; X. F. Wang et al., 2004). Details of culture and assays confirming onset of senescence are described in the Appendix S1, *Materials and Methods*.

## 3 RESULTS

### 3.1 Age‐related global histone loss in mouse RPE

**FIGURE 1:**
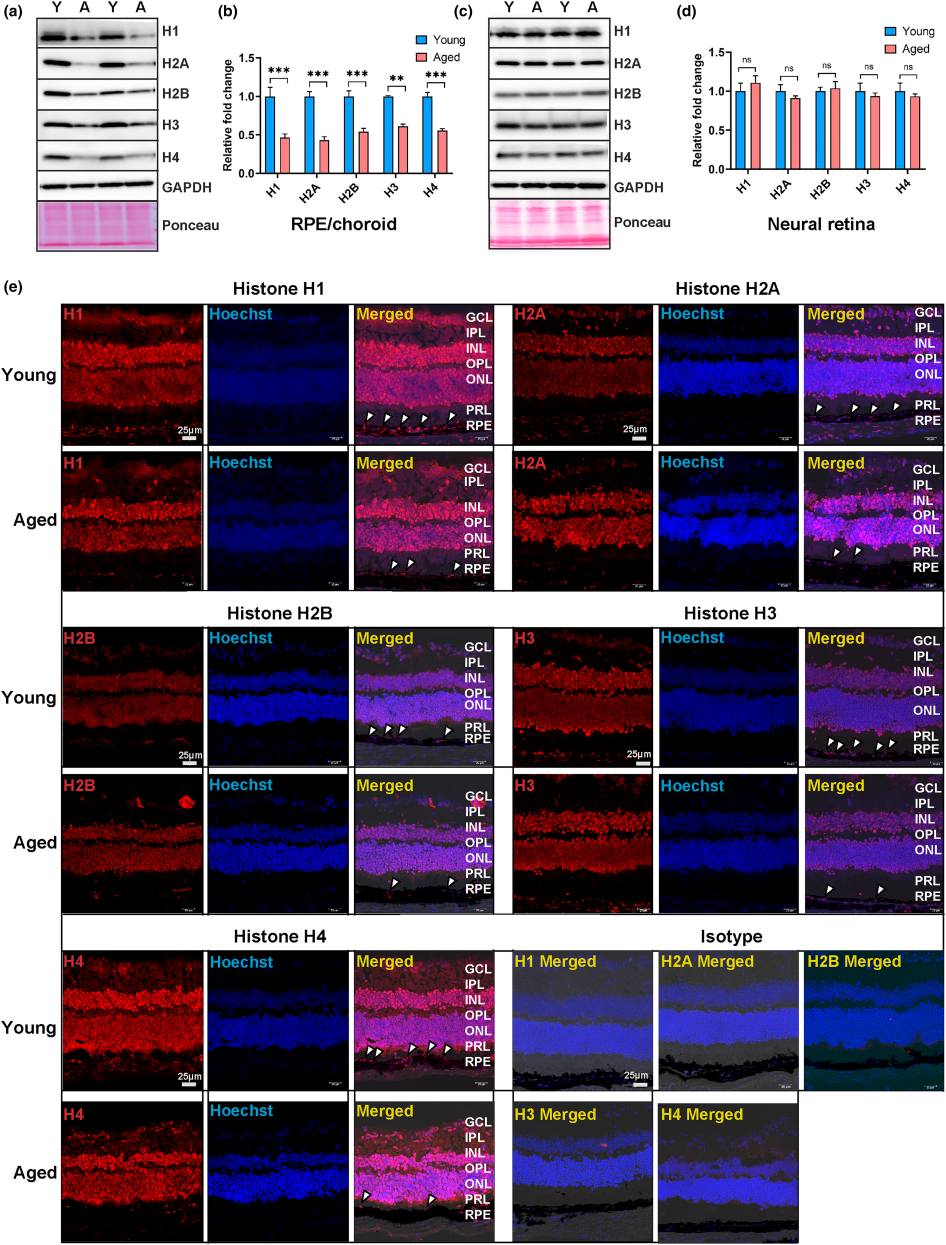
Loss of histone protein in aged mouse RPE/choroid. (a) Representative images of Western blot analysis of histones H1, H2A, H2B, H3, and H4, along with GAPDH loading control, in RPE/choroid from young and aged mice (*n* = 6–8 per group). Ponceau‐S stained blot confirmed equal loading across samples. (b) Densitometry analysis showed a significant loss of core and linker histones, normalized to GAPDH, in RPE/choroid of aged mice. (c) Representative Western blots of neural retina from the young and aged mice (*n* = 6 per group) of all five histones along with GAPDH loading control. Ponceau‐S stained blots confirmed equal loading across samples. (d) Histone levels were quantified by densitometry, revealing no significant changes between young and aged mice. The statistical significance of densitometry data was determined by Mann–Whitney *U* test (*p < 0.05, **p < 0.01, ***p < 0.001, ns: nonsignificant). The averages (mean ± SEM) of the three independent experiments are shown. (e) Representative fluorescence images of retinal cross‐sections of young and aged mice showing the distribution of histones across different layers of the retina. Intense staining for H1, H2A, H2B, H3, and H4 histones (red) was found across the neural retina in young and aged RPE. Nuclei are stained with Hoechst (blue) in both the young and aged retina. Merged images of overlapping red and blue channels with brightfield showed the localization of histones in the RPE layer. The RPE layer of aged mice showed weak staining for all histones (white arrows) compared to the young mice. The isotype panel showed no immunostaining and served as a negative control. GCL, ganglion cell layer; IPL, inner plexiform layer; INL, inner nuclear layer; OPL, outer plexiform layer; ONL, outer nuclear layer; PRL, photoreceptor layer, RPE; retinal pigment epithelium. Scale bar: 25 μm.

Cellular aging is accompanied by an altered histone profile, including changes in histone levels, accumulation of specific histone variants, and dynamic modifications in histone PTMs. These histone signatures play a crucial role in modulating the structure and organization of chromatin. To investigate the potential impact of age‐related changes on histone levels in RPE and retina, we prepared tissue extracts from RPE/choroid and neural retina and compared the protein levels of histones H1, H2A, H2B, H3, and H4 between young and aged mice (*n* = 6–8 samples per group). We observed a global reduction of histone proteins H1, H2A, H2B, H3, and H4 in RPE/choroid as a consequence of aging (Figure 1a). Densitometric analysis revealed a substantial depletion of histone proteins ranging from ~40% to 55% in aged RPE/choroid (Figure 1b). By contrast, examination of the neural retina from young and aging mice (*n* = 6 samples per group) showed no significant changes in histone levels (Figure 1c, d). Immunostaining of frozen eye sections confirmed decreased levels of all core and linker histone proteins in the RPE layer in aged mice (Figure 1e). Importantly, this finding was unique to the RPE, as histone expression was unchanged in the neural retina in young and aged mice. Histone proteins were distributed throughout the neural retina, displaying intense staining in the ganglion cell layer (GCL), inner nuclear layer (INL), and outer nuclear layer (ONL). Isotype controls exhibited no staining (Figure 1e). At high magnification (63x), confocal microscopy confirmed decreased expression of histones in aged RPE compared to young RPE (Figure S1). Our findings suggest that in the mouse eye, there is a significant age‐related loss of histone expression in the RPE. By contrast, histone expression in the aging neural retina remains robust and consistent.

### 3.2 Transcriptional downregulation of histones and HLB components in aged mouse RPE

**FIGURE 2:**
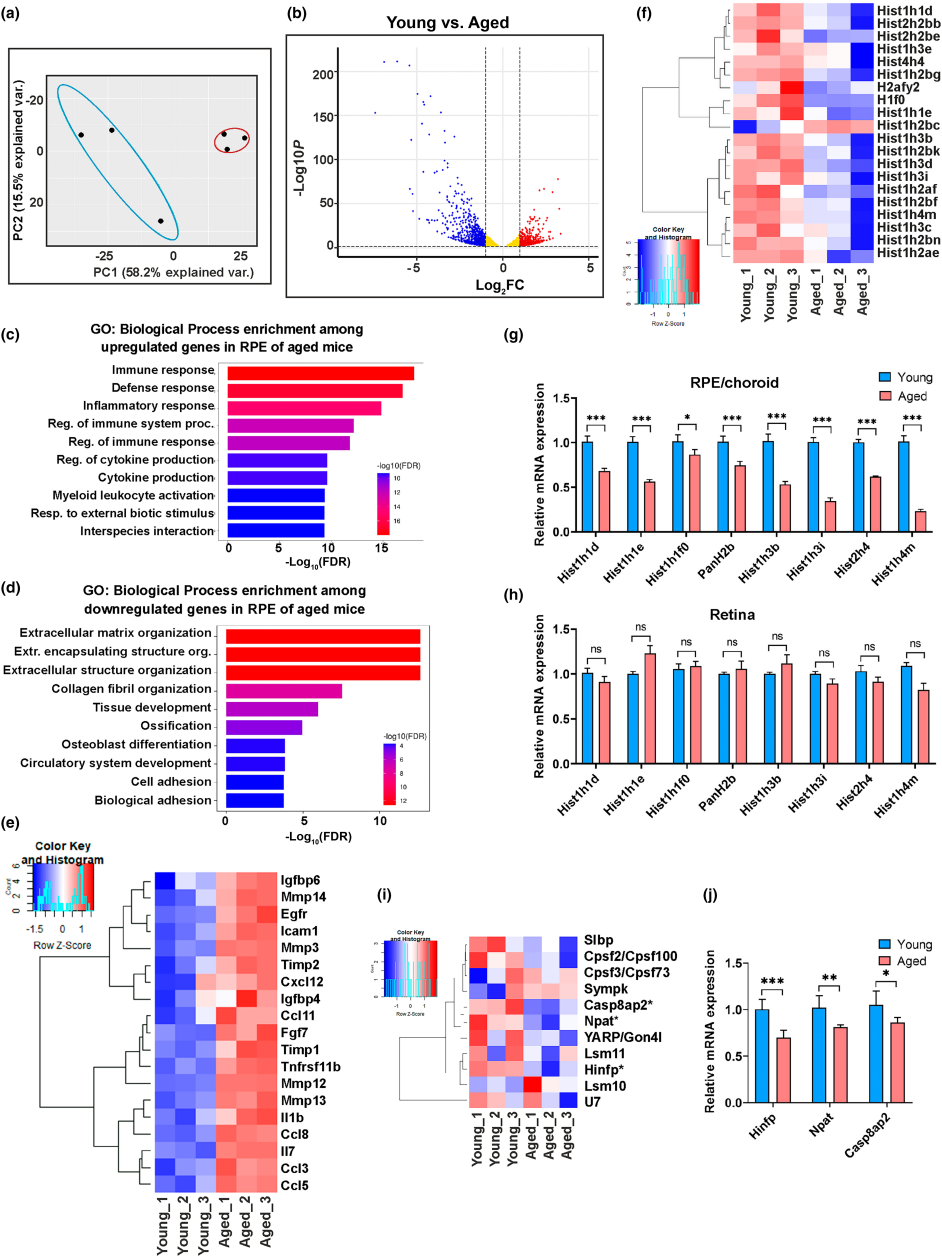
Transcriptome analysis and histone profiling in young and aged mouse RPE/choroid. (a) PCA plot generated using the whole transcriptome dataset showed distinct clustering of young and aged RPE/choroid along the first principal component (PC1), accounting for the highest total variance (58.2%). Blue and red ellipses represent young and aged mice RPE, respectively. Data are from three biologically independent replicates. (b) Volcano plots showing differentially expressed genes (DEGs) as log_2_ fold change (FC) between young and aged mice RPE along the x‐axis and the y‐axis shows the statistical significance of the differences (log_10_ adjusted *p*‐value). Red dots represent the upregulated genes, and blue dots represent the downregulated genes in young RPE. The horizontal dotted gray line represents adj. *p*‐value <0.05, and the vertical dotted gray lines represent log_2_FC values of −1 and + 1. (c and d) The top 10 enriched terms from GO analysis of top 100 up‐ (c) and down‐regulated (d) genes in young versus aged mice RPE/choroid. The x‐axis represents the ‐log_10_ scale for adjusted *p*‐value of each GO term. (e) Hierarchical clustering and heatmap analysis of selected SASP markers in the RPE/choroid of young and aged mice. Blue to red represents low to high gene expression. (f) Hierarchical clustering and heatmap of 20 histone variants found to be significantly downregulated (adj *p*‐value <0.05) in aged RPE/choroid. Blue to red represents low to high gene expression. (g and h) Relative expression levels of selected histone genes validated by qPCR in mouse (*n* = 5) RPE/choroid (g) and neural retina (h). (i) Hierarchical clustering and heatmap analysis showed the expression profile of HLB components. *Represent genes that are significantly downregulated in aged RPE. (j) qPCR analysis of *Hinfp*, *Npat*, and *Casp8ap2* genes in young and aged RPE/choroid (*n* = 4–6). GAPDH was used for normalization, and statistical analysis was performed using the Mann–Whitney *U* test (*p < 0.05, **p < 0.01, ***p < 0.001) in g, h, and j. Results presented as mean ± SEM.

To investigate if age‐related loss of histones is evident at the transcriptional level, we performed transcriptomic profiling of RPE/choroid tissues from young (2–3 months) and aged mice (20–24 months). Since most histone transcripts are non‐poly(A) mRNAs, we sequenced six rRNA‐depleted libraries (*n* = 3 per group) without poly(A) enrichment to profile the expression of histone mRNAs (Figure S2a; GEO accession number GSE236221). The average sequencing depth of the libraries was 40.4 million, and a total of 33,924 transcripts were mapped to the mouse reference genome. Principal component analysis (PCA) of transcriptome data revealed distinct clusters for young and aged mice along PC1, accounting for 58.2% of the variance, indicating age as the primary driving factor (Figure 2a). DESeq2 analysis of read counts revealed significant expression changes in 3949 genes between young and aged RPE/choroid (adjusted *p*‐value <0.05). The magnitude of differential gene expression was visualized using a volcano plot (Figure 2b), representing 1849 upregulated genes and 2100 downregulated genes in young RPE compared to aged RPE. To obtain a comprehensive view of the biological processes represented in these significantly altered genes, Gene Ontology (GO) term enrichment analysis was performed using the top 100 upregulated and downregulated genes (Figure 2c, d; Tables S1 and S2). GO enrichment of genes upregulated in aged RPE revealed an elevated expression of genes associated with immune response and inflammation, whereas a majority of genes downregulated in aged RPE were mainly related to extracellular matrix organization and organ and tissue development. Previous studies showed that aged tissues exhibit a low‐grade inflammatory state and increased SASP expression (Coppe et al., 2010). Therefore, we examined a panel of senescent markers from our transcriptome data and found upregulation of all these markers in aged RPE tissue (Figure 2e).

We identified a collection of 114 mouse histone gene variants from Mouse Genome Informatics (Blake et al., 2021) and MS_HistoneDB (El Kennani et al., 2017) databases and compared them with our transcriptome data. Comparative analysis of these 114 variants revealed significant differential expression (adjusted *p*‐value <0.05) of 20 histone genes between young and aged mice RPE/choroid (Table S1). Hierarchical clustering and heatmap analyses showed age‐associated clustering and downregulation in most histone genes in aged RPE/choroid (Figure 2f). Among the examined histones, three H1, three H2A, six H2B, five H3, and two H4 isoforms showed significant downregulation in aged RPE/choroid. Conversely, one unique histone gene, *Hist1h2bc*, known to be expressed as polyadenylated mRNA in certain differentiated cells, was upregulated in aged mouse RPE/choroid (Figure 2f) (Lyons et al., 2016). To validate our RNA‐seq data, we performed qPCR analysis on eight selected *Hist1* and *Hist2* genes in aged and young mice (*n* = 5 per group) RPE/choroid samples. These results corroborated our RNA‐seq data with target histone genes significantly downregulated in aged RPE/choroid (Figure 2g), while neural retina showed no significant change (Figure 2h). Collectively, these findings strongly suggest that the loss of histones is a prominent characteristic of aging mouse RPE/choroid.

We next examined the expression profile of histone regulatory genes in young and aged RPE/choroid. RNA‐Seq libraries were prepared from rRNA‐depleted and poly(A)‐selected young and aged mice RPE samples to include protein‐coding poly(A)‐tailed mRNAs (Figure S2a). This method is known to reduce the noise rate by excluding the pre‐mRNAs (L. Chen, Yang, et al., 2020). The RNA‐seq data from ribo‐depleted, poly(A)‐selected samples (GEO accession number GSE236221) of young and aged RPE/choroid (*n* = 3 per group) were subjected to differential expression analysis using the DESeq2 Bioconductor package. There were 4231 significantly regulated genes (adjusted *p*‐value <0.05) including 1878 upregulated and 2353 downregulated genes (top 100 genes are listed in Tables S3 and S4). Differential gene expression showed a strong correlation (Pearson's *R* = 0.8) between rRNA‐depleted and poly(A)‐enriched libraries (Figure S2b). GO analysis of the top 100 up‐ and down‐regulated genes from the poly(A)‐enriched libraries revealed that aged RPE harbored increased expression of immune‐inflammatory mediators, coupled with downregulation of genes critical to extracellular matrix organization and homeostasis (Figure S2c).

In further transcriptomic analyses of histone regulatory genes, we discovered that expression of requisite HLB components including *Hinfp*, *Npat*, and *Casp8ap2*, were significantly decreased in aged RPE/choroid (Figure 2i). Validation by qPCR again showed downregulation of *Hinfp* (0.70‐fold change), *Npat* (0.81‐fold change), and *Casp8ap2* (0.86‐fold change) expression in aged mouse RPE/choroid (Figure 2j). Thus, the reduction in histones observed in aging RPE may be attributed to the downregulation of HLB complex components revealing a novel therapeutic target for age‐related retinal diseases.

### 3.3 *HINFP* knockdown induced global histone loss and senescence in human RPE cells

**FIGURE 3:**
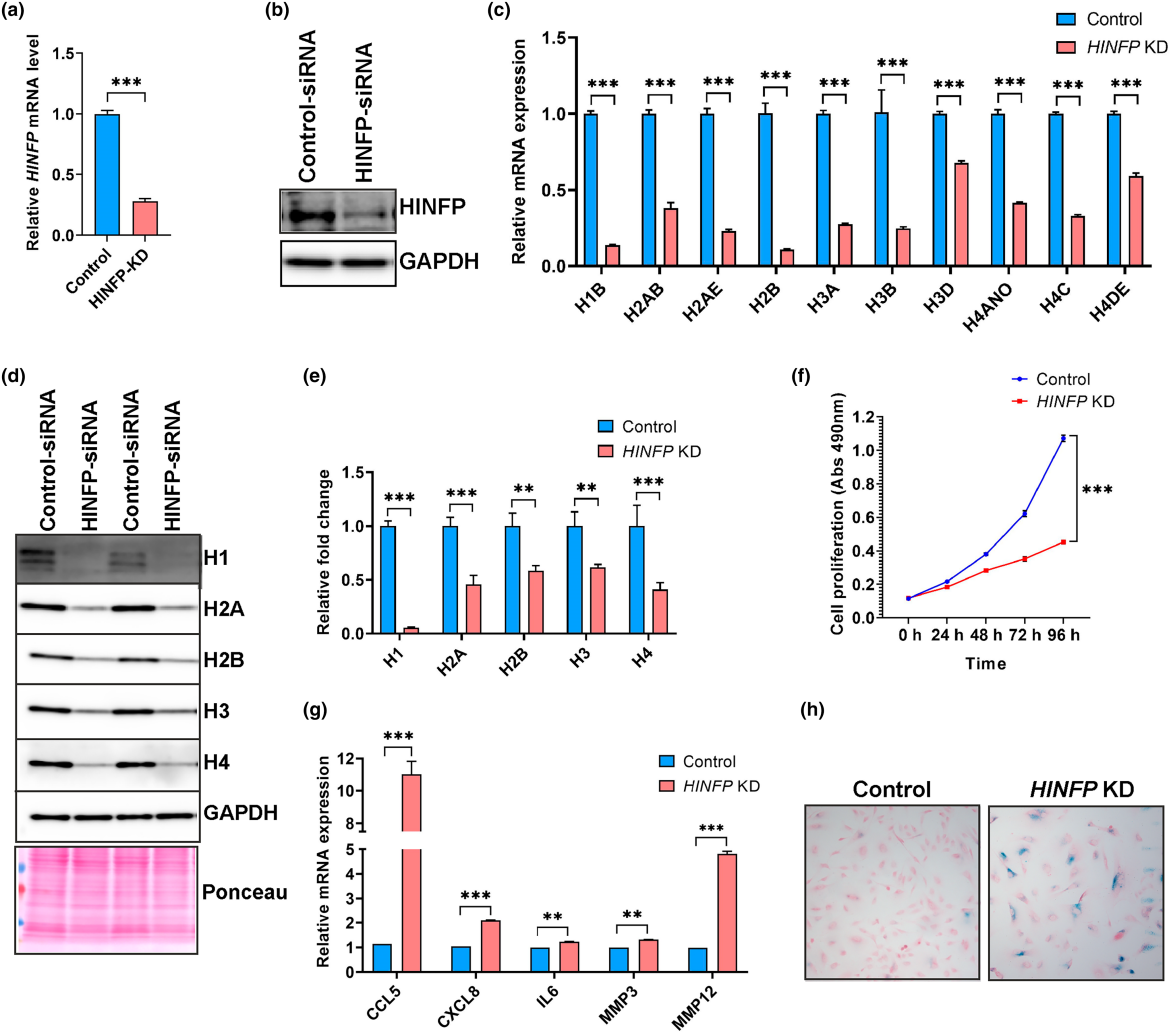
Reduced histone levels and accelerated hRPE senescence after *HINFP* knockdown. (a) Evaluation of relative *HINFP* level by qPCR in hRPE cells treated with control‐ and HINFP‐siRNAs. (b) Western blot of *HINFP* siRNA‐treated hRPE cells showed a significant knockdown of HINFP compared to scramble siRNA‐treated controls. (c) qPCR analysis of selected histone isoforms in control and *HINFP* KD hRPE cells (*n* = 3 isolates). (d) Representative images of Western blot analysis of histones H1, H2A, H2B, H3, H4, and GAPDH loading control in *HINFP* KD and control hRPE cells. Ponceau‐S stained blot indicated equal sample loading. (e) Densitometry analysis showed a significant loss of core and linker histones in hRPE cells treated with *HINFP* siRNA compared to scramble siRNA‐treated controls (*n* = 3 isolates). (f) MTT assay was used to measure cell proliferation in control and *HINFP* KD hRPE cells. The results are shown as the mean ± SD of three independent experiments performed in triplicate. (g) SASP markers were analyzed by qPCR analysis in control and *HINFP* KD hRPE cells. (h) Representative images of control and *HINFP* KD hRPE cells showing cellular senescence by SA‐β‐Gal staining (blue) and eosin counterstain (red). GAPDH was used for normalization and results were presented as mean ± SEM in a, c, e, and g. Statistical analysis was performed using either Mann–Whitney *U* test as in c, e, or unpaired *t*‐test as in a, f, and g (*p < 0.05, **p < 0.01, ***p < 0.001, ns: nonsignificant).

With our discovery of core and linker histone deficiency in the aged mouse RPE, we next investigated whether histone depletion could initiate the early onset of aging and senescence in primary hRPE cells in vitro. The challenge of studying histone depletion arises from the vast number of genes encoding each histone protein. To overcome this challenge, we targeted HINFP, a key component of the HLB complex involved in the regulation of histone transcription. The rationale for targeting HINFP stems from the critical role played by HINFP as a transcriptional endpoint factor and is recruited directly to specific recognition sites of histone promoters (Miele et al., 2005; Mitra et al., 2003). We utilized siRNA‐mediated knockdown (KD) of *HINFP* in early passage hRPE cells (P3‐4) and confirmed the downregulation of *HINFP* expression by qPCR and Western blot analysis (Figure 3a, b). *HINFP* KD in hRPE cells resulted in significant downregulation of all assayed core and linker histone isoforms (Figure 3c). Notably, protein levels of core and linker histones displayed a substantial downregulation ranging from 40% to 90% in *HINFP* KD cells with histone H1 exhibiting the most prominent reduction (Figure 3d, e). To explore the impact of *HINFP* KD on cell proliferation, we performed MTS assays which exhibited significantly reduced proliferation of hRPE cells following *HINFP* KD (Figure 3f). *HINFP* KD cells exhibited early signs of aging and senescence with upregulation of SASP markers (*CCL5*, *CXCL8*, *IL6*, *MMP3*, *and MMP12*) (Figure 3g) and harbored significantly increased numbers of senescence‐associated β‐galactosidase (SA‐β‐Gal) positive cells compared to the control group (Figure 3h). These data establish that HINFP is the master regulator controlling the transcription of all histones in RPE and that HINFP‐mediated histone loss induced accelerated aging and senescence in RPE cells in vitro as evidenced by reduced proliferation, upregulation of SASP markers, and an increased number of SA‐β‐Gal positive cells.

### 3.4 Downregulation of histone expression with replicative aging of hRPE cells

**FIGURE 4:**
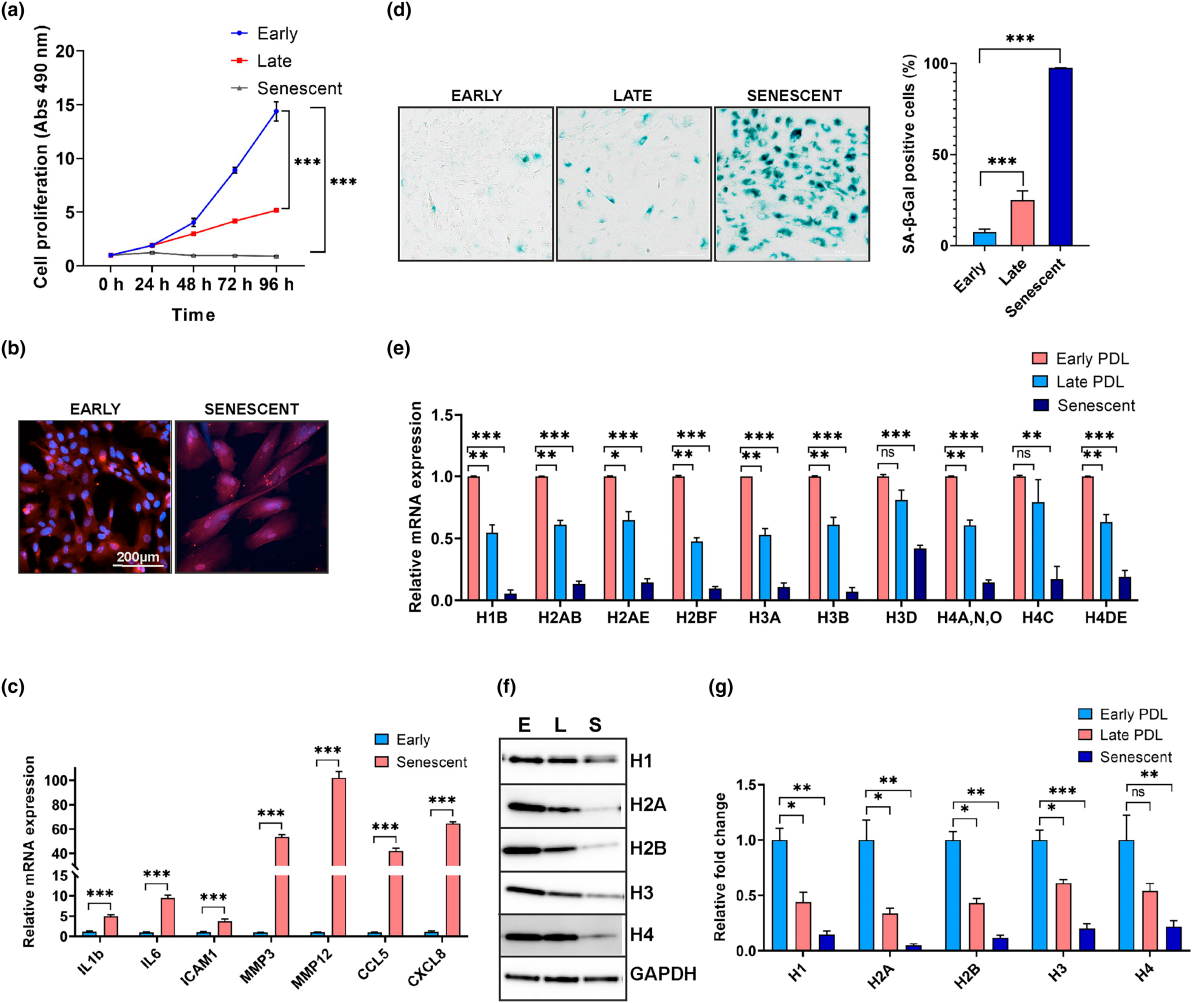
Evaluation of histones in the replicative senescence model of human RPE. (a) Cell proliferation rates of hRPE isolates (*n* = 3) in early (e), late (L), and senescent (S) cells were measured using MTT assay. (b) Representative fluorescence images showed localization of RPE65 (Red) in the cytoplasm of hRPE cells with Hoechst‐positive (Blue) nucleus in both E and S cells. (c) SASP markers were analyzed by qPCR and normalized with GAPDH. (d) SA‐β‐Gal activity in hRPE cells from E, L, and S passages. SA‐β‐Gal cells in three random fields were quantified with results expressed as a percentage of stained cells in total cells counted. (e) qPCR analysis of histone isoforms from E, L, and S hRPE cells (*n* = 3). (F and G) Western blot and densitometry analysis showed progressive loss of all five histones in L and S cells compared to E hRPE cells. GAPDH is used for normalization in c, e, and g. Data are presented as the mean ± SEM in a, c, d, e, and g. Statistical significance was assessed using unpaired t‐test (*p < 0.05, **p < 0.01, ***p < 0.001, ns: nonsignificant) in a, c, d, e, and g.

Passage‐mediated replicative aging of cells has been widely used as an in vitro model for aging (X. F. Wang et al., 2004). Here, we studied the effects of replicative aging on histone expression in primary hRPE cells. Early passage (P3‐P4) hRPE cells (*n* = 3 isolates) were grown to 70%–80% confluence, repeatedly trypsinized, and subcultured at a 1:4 ratio, until the cells reached replicative exhaustion. The population doubling level (PDL) for each passage was estimated by taking two PDLs per passage (Matsunaga et al., 1999), and by PDL 46–50, all hRPE isolates ceased cell division and entered senescence. To assess the impact on cell proliferation, the MTS assay was performed using hRPE cells (n = 3) from early (PDL 10–12), late (PDL 34–40), and senescent (last PDL) cells at various time points (0, 24, 48, 72, and 96 h). Comparing hRPE cells at later PDLs to early PDLs, we observed that cells at late PDLs had decreased cell proliferation while senescent cells showed no proliferation (Figure 4a). Moreover, senescent cells exhibited markedly different morphology with flattened and elongated cells compared with early PDLs, yet maintained their RPE phenotype as they stained positive for RPE65, a classical RPE marker (Figure 4b). The onset of senescence in hRPE cells was accompanied by the increased secretion of SASP factors, including proinflammatory cytokines (*CXCL8* and *CCL5*), interleukins (*IL1b* and *IL6*), and extracellular matrix components (*ICAM1*, *MMP3*, and *MMP12*) (Figure 4c). Furthermore, SA‐β‐Gal staining of early, late, and senescent RPE cells showed a substantial increase in SA‐β‐Gal activity in senescent cells corresponding to increased lysosomal enzymes (Figure 4d). The percentage of SA‐β‐Gal positive blue stained cells was ~7% in the early passage and this dramatically increased to ~25% in the late passage and >95% in senescent cells (Figure 4d). Analysis of histone expression across early, late, and senescent cells revealed a distinct signature of downregulation for various histone isoforms (Figure 4e). Western blot and densitometry analysis revealed that all core and linker histones progressively declined in aging cells with a significant loss of all five histones in senescent cells (Figure 4f, g). Our results demonstrated a gradual loss of histone expression and acquisition of SASP phenotype by hRPE following replicative aging highlighting an intricate interplay between histone expression profiles and cellular senescence in hRPE cells.

### 3.5 Histone expression in human RPE decreases with age

**FIGURE 5:**
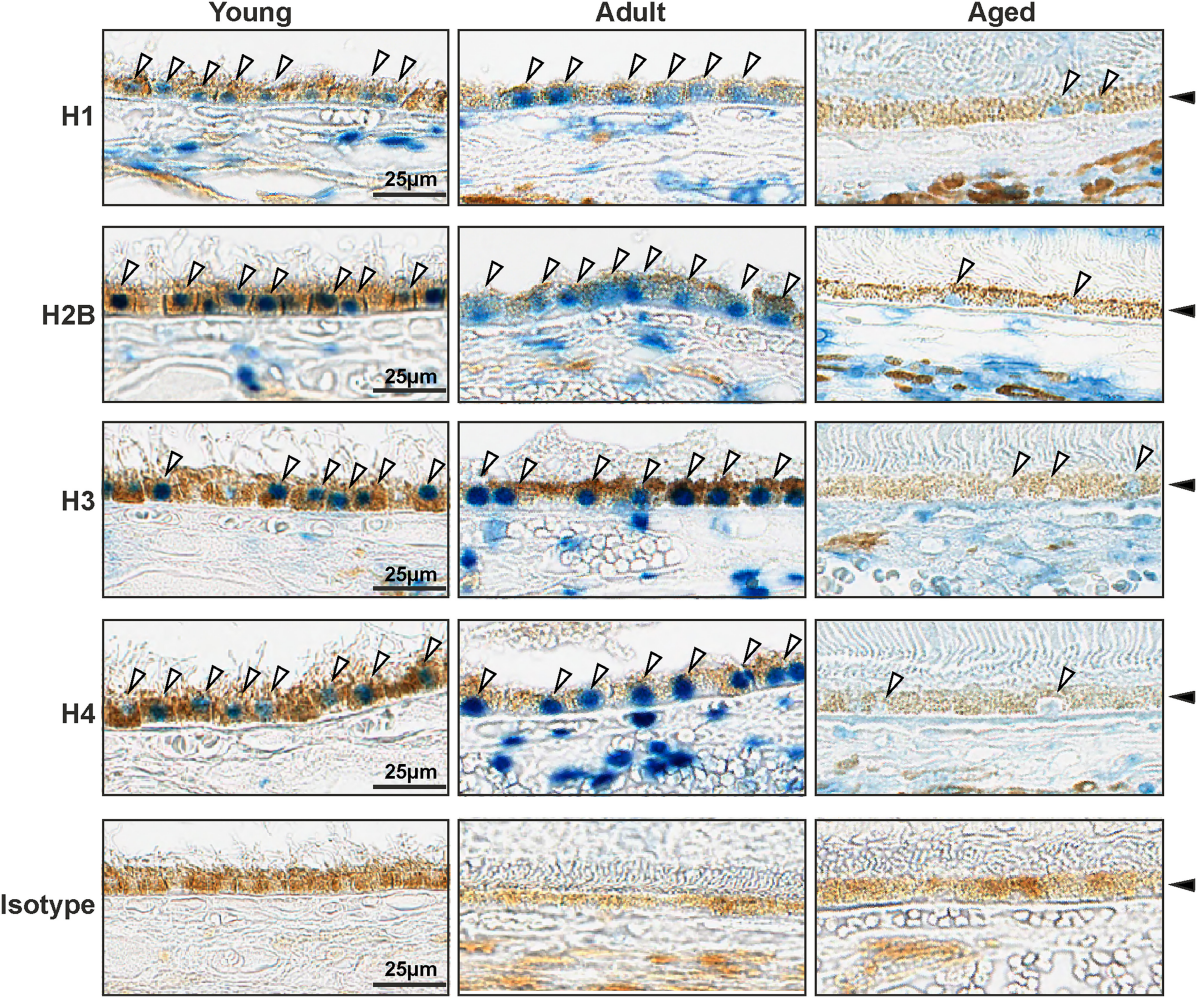
Immunohistochemical analysis of histone expression in the human RPE tissue. (a) Immunostaining for histone H1, H2B, H3, and H4 on FFPE human eye sections (*n* = 2 young; *n* = 3 adult; *n* = 3 aged, representative of 5–13 y.o., 36–48 y.o., and 60–82 y.o. eyes shown) demonstrated markedly decreased expression of histones (white arrows) in the RPE layer (solid black arrow) of aged compared to young and adult donor eyes. Isotype controls showed no staining. Scale bar 25 μm.

Next, we sought to correlate our findings of histone depletion in aged mouse RPE with human RPE. We examined formalin‐fixed paraffin embedded (FFPE) human sections from young (5–13 y.o.), adult (36–48 y.o.), and aged (60–82 y.o.) normal donor eyes to determine expression levels of histones confirmed by qPCR validation of mouse RPE/choroid transcriptome data (H1, H2B, H3, and H4). Immunohistochemistry for these histones revealed a significant loss of expression in the RPE of aged tissues compared to young and adult RPE with isotype labeling revealing no detectable immunostaining (Figure 5). Neural retinal expression of histones could not be rigorously examined in human sections due to separation of the retinal layers on the precious young human eyes. Collectively, the data establish for the first time that histone expression is significantly decreased in the aged human RPE.

### 3.6 Aging impacts global histone acetylation in the mouse RPE

**FIGURE 6:**
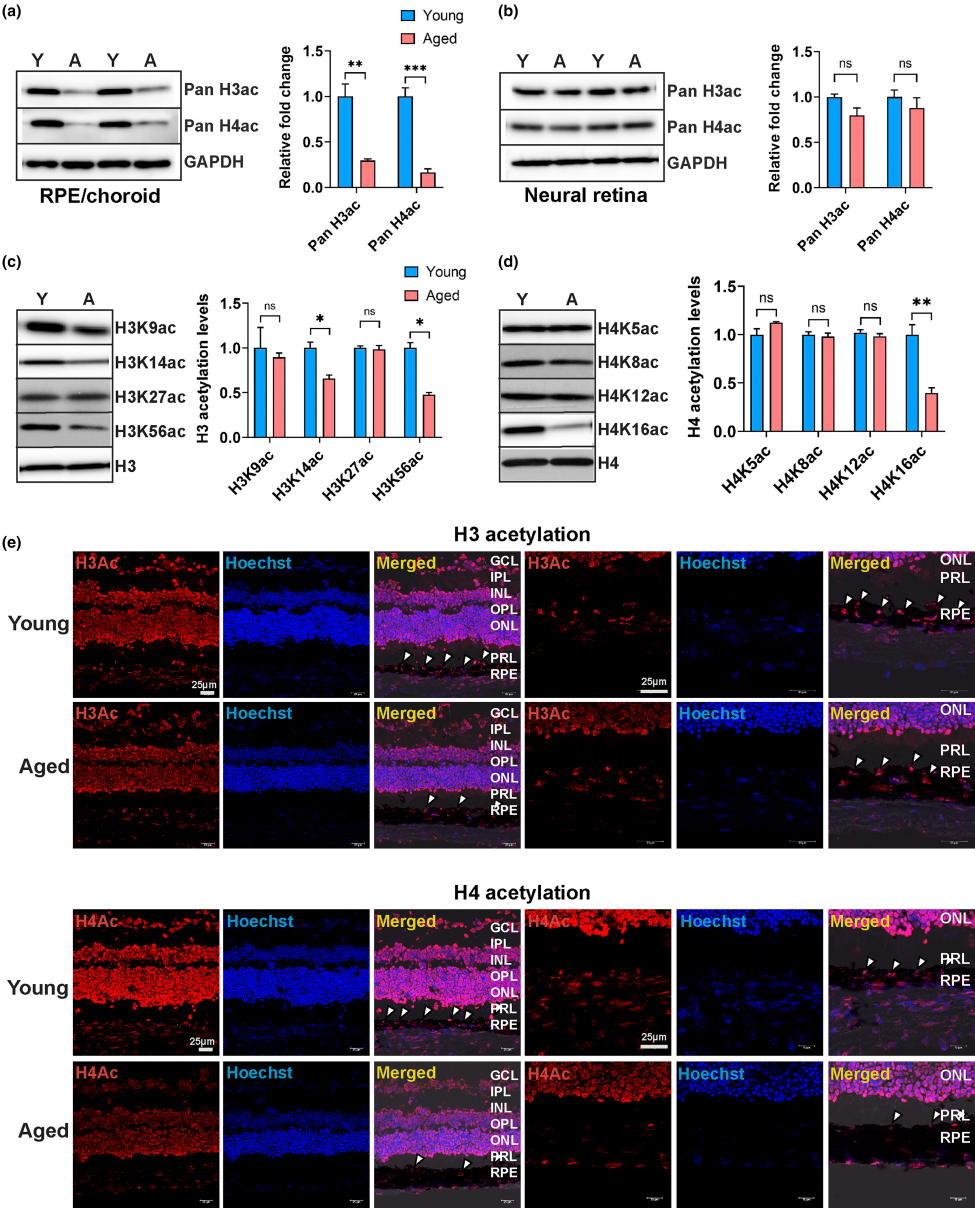
Acetylated histone H3 and H4 levels in RPE/choroid of young and aged mice. (a) Representative Western blots of RPE/choroid cell lysates from young and aged mice showing pan‐acetyl H3, pan‐acetyl H4, and GAPDH loading control. Densitometry values for H3ac and H4ac showed significant depletion of acetylation in aged RPE/choroid (*n* = 4–6 per group). (b) Western blots of pan‐acetyl H3, pan‐acetyl H4, and GAPDH from retinal cell lysates of young and aged mice. Densitometry values for H3ac and H4ac showed no significant changes in acetylation levels between young and aged neural retinas (*n* = 4–6 per group). (c and d) Western blots of histone extracts using acetyl‐specific antibodies show the acetylation status of H3K9/K14/K27/K56 (c) and H4K5/K8/K12/K16 (d) in RPE/choroid of young and aged mice. Densitometry analysis of Western blots for specific acetylation marks on H3 and H4 showed a significant loss of acetylation at H3K14/K56 and H4K16 marks (*n* = 3–4 per group). (e) Representative fluorescence images of H3 and H4 acetylation in RPE of young and aged mice. Staining for pan‐H3ac and ‐H4ac (red) was stronger in the RPE (white arrows) of young mice compared to aged mice. Nuclei are stained with Hoechst (blue) in both young and aged retina. Merged images of overlapping red and blue channels with brightfield showed localization of H3ac and H4ac in the RPE layer. Scale bar: 25 μm. Data were normalized to GAPDH (in a and b), H3 (in c), and H4 (in d). Statistical testing was performed using the unpaired *t*‐test (*p < 0.05, **p < 0.01, ***p < 0.001, ns: nonsignificant) and data were presented as mean ± SEM.

Given the depletion of histones in aged mouse RPE, we then evaluated global histone acetylation levels, as acetyl‐histone modifications are a critical PTM for epigenetic control of transcription and silencing. Acetyl marks on histones H3 and H4 are more conserved and stable compared to acetyl marks on histones H2A and H2B (Talbert & Henikoff, 2017). Thus, we measured Pan‐acetyl H3 (H3K9ac + H3K14ac + H3K18ac + H3K23ac + H3K27ac) and pan‐acetyl H4 (H4K5ac + H4K8ac + H4K12ac + H4K16ac) levels in whole cell lysates from young and aged mouse RPE/choroid and neural retina by Western blot (Figure 6a, b). Densitometry analyses revealed significantly decreased pan‐acetyl H3 and ‐acetyl H4 levels in aged mouse RPE/choroid (Figure 6a), whereas pan‐acetyl H3 and ‐acetyl H4 levels in the neural retina of young and aged mice remain unchanged (Figure 6b).

To further quantify transcriptionally important specific acetyl marks on H3 and H4, histone extracts were prepared from RPE/choroid (*n* = 3–4 per group) of young and aged mice followed by immunodetection using Western blot. For these assays, we loaded an equal amount of histone extract which normalized the effect of the observed H3 and H4 losses in aged RPE and allowed H3 and H4 to serve as a loading control. Western blot and densitometry revealed a significant decrease in H3K14ac, H3K56ac, and H4K16ac marks in aged mouse RPE/choroid while no observable change in acetylation levels was observed at H3K9ac, H3K27ac, H4K5ac, H4K8ac, and H4K12ac (Figure 6c, d). Immunofluorescence studies of retinal sections of young and aged mice confirmed the loss of pan‐acetyl H3, pan‐acetyl H4 (Figure 6e), and an important lysine acetyl‐histone mark, H4K16 (Figure S3) identifying a novel signature of histone acetylation in aged mouse RPE.

Next, we interrogated the expression of histone acetyltransferases (HATs) and histone deacetylases (HDACs) which are responsible for the addition and removal of acetyl groups on histone proteins, respectively. We compared a list of HATs and HDACs from our transcriptome data (Figure S4a,b) and discovered that expression levels of HATs and HDACs are not significantly altered by aging except for *Sirt4*, a mitochondrial deacetylase involved in deacetylating nonhistone substrates (Zhu et al., 2014). Furthermore, we conducted fluorescence‐based HDAC activity assays using aged and young mouse RPE/choroid tissue lysates and found no significant difference in HDAC activity between the groups (Figure S4c). These findings suggest that expression and activity of HDACs remain unaffected by aging in RPE/choroid. Together, our study provides substantial evidence linking histone loss and hypoacetylation of histones in the aged mouse RPE and sheds light on the potential epigenetic mechanisms underlying age‐related loss of RPE cell viability and function.

## 4 DISCUSSION

Epigenetic studies of aging mammalian tissues and other cellular models have revealed a common theme including dysregulated histone expression profiles with increased variant levels, altered histone PTM marks, and evidence of significant chromatin remodeling (Pal & Tyler, 2016; Sen et al., 2016). Tight regulation of histone expression is essential to maintain chromatin structure and appropriate gene expression profiles critical to cell viability and function. In the current study, we examined the expression profile of core and linker histones in the RPE/choroid of young and aged mice, and our results demonstrated an age‐related depletion of all five histones in the RPE (Figure 1a, b, e, and Figure S1). Histone loss with age is observed across various organisms, from yeast to mammals, underscoring its significance in aging (Feser et al., 2010; Kim et al., 2021; Liu et al., 2013). A previous study in aging quiescent muscle stem cells showed a reduction of approximately 40% in histone expression (Liu et al., 2013), comparable with our observations of ~40%–55% histone loss in aging RPE (Figure 1b). A subsequent study of aged human proliferating naive CD4+ T‐cells also showed reduced histone expression causing a delayed cell cycle progression through the S‐phase (Kim et al., 2021). The postmitotic nature of the RPE layer in the eye makes it a valuable model for studying the impact of histone level reduction on genome organization and cellular pathways in terminally differentiated cells. Importantly, downregulation of histones was unique to the aging RPE, as the neural retina maintained a robust expression of all the histones interrogated in aged mice. The RPE provides critical trophic support to the neural retina via visual cycle processing, nutrient exchange, and phagocytosis. Given the multiple highly demanding cell functions, it is very susceptible to aging, as demonstrated by recent single‐cell transcriptomic data from young and aged eyes of nonhuman primates (S. Wang et al., 2021). Our data suggest that histone deficiency in the aging RPE may be an initiating event for loss of RPE cell viability and declining retinal function with age and other age‐related retinal diseases.

It is critical to note that the neural retina is a complex, multilayered structure, and our current study did not include an analysis of histone gene expression levels in individual layers of the neural retina. The neural retina mainly consists of three distinct cellular populations, retinal ganglion cells (RGCs), interneurons, and the light‐sensitive photoreceptors (PRs). The PR layer relies heavily on the RPE for structural and functional integrity. It is essential to acknowledge that RPE atrophy precedes PR cell loss in AMD, as demonstrated by previous studies (L. Chen, Messinger, et al., 2020; Christenbury et al., 2013). However, the current study focused on age‐related cellular senescence of the RPE and did not delve into the effects of RPE senescence on PR cell viability. This aspect will be investigated in our future work.

Canonical histones are synthesized during the S‐phase of the cell cycle and rapidly transferred onto the growing DNA strand. By contrast, histone variants are synthesized throughout the cell cycle and can replace the canonical histones, thereby driving chromatin differentiation (Henikoff & Smith, 2015). Multiple studies have reported the substitution of canonical histones by histone variants during aging leading to changes in the epigenetic landscape (Kreiling et al., 2011; Stefanelli et al., 2018). The histone isoform variant H3.3 is known to replace canonical histones H3.1 and H3.2 during aging, and accumulation of H3.3 was observed in aged mouse neurons and other tissues such as the liver, kidney, brain, and heart (Maze et al., 2015; Tvardovskiy et al., 2017). In our study, we did not observe significant changes in the expression of H3.3 variant genes (*H3f3a* and *H3f3b*) between young and aged RPE/choroid (Table S1). Other histone variants like *H2afy2*, *Hist1h2bk*, and *H1f0* were reported to accumulate in various aging cells and animal models (Contrepois et al., 2021; Kreiling et al., 2011; Sekeri‐Pataryas & Sourlingas, 2007; Zhang et al., 2005). Yet, our data demonstrated lower expression of these isoforms in the aged RPE (Figure 2f). In light of previous studies, our results strongly suggest that any changes in histone abundance and nucleosome composition associated with aging occur in a cell type‐specific manner and that the aged RPE develops a unique histone expression signature. Aged RPE also develops increased SASP secretion and a propensity for pro‐inflammatory gene expression which are known factors of tissue aging (Figure 2c,e). Lastly, we performed immunostaining experiments on aged and young human eyes to examine core and linker histone protein levels in the posterior segment. These data clearly demonstrated decreased expression of histones in the RPE of aged individuals corroborating the results from our aged mouse model (Figure 5).

Histone proteins typically have highly stable half‐lives spanning from several days to months depending on the rate of cell proliferation and the differentiation status of the cells (Mathieson et al., 2018). We investigated the factors influencing histone gene transcription to understand potential molecular mechanisms underlying histone depletion in aged RPE. Our analyses focused on the proteins central to the organization of HLBs and identified that *Hinfp*, *Npat*, and *Casp8ap2* were significantly downregulated in aged RPE/choroid (Figure 2i). Previous studies have established that the precise organization of the transcriptional machinery regulating histone gene expression depends on the recruitment and functional fidelity of the cyclin‐E/CDK2/NPAT/HINFP pathway at the HLBs (Miele et al., 2005). Specifically, HINFP has been identified as a critical player in this pathway and directly binds to the histone gene promoter to mediate the recruitment of NPAT to the HLB complex (Ghule et al., 2014; Miele et al., 2005). Therefore, we predicted that targeting this upstream regulator of histone biosynthesis would singularly disrupt the vast array of histone genes in RPE. The hRPE *HINFP* KD cell lines were deficient in all core and linker histones and exhibited reduced cell proliferation (Figure 3c–f). Other studies have reported that the loss of HINFP and NPAT can lead to the deregulation of histone gene expression which has many implications on cell cycle progression and viability (Di Fruscio et al., 1997; Ghule et al., 2014). Nevertheless, such an extensive impact of knockdown of *HINFP* on the expression of all histones has yet to be demonstrated in human cells. While siRNA‐mediated knockdown of *HINFP* in HEK‐293 cells only decreased expression of H4 and two variants of H1, H1.X, and H1.0, the same study showed Drosophila carrying *Hinfp* mutants experienced a reduction of approximately 50% in core histones H2A, H2B, H3, and H4 along with significant depletion of histone H1 by 95% (Nirala et al., 2021). Several studies in mouse models suggested Hinfp is mainly involved in the regulation of the histone H4 genes and that Hinfp‐mediated positive regulation of histone H4 is essential for cell cycle progression in human embryonic stem cells and early mouse embryonic development (Ghule et al., 2008; Miele et al., 2005; Mitra et al., 2003; Xie et al., 2009). Our data provides the first in vitro evidence of *HINFP* globally regulating the expression of histones in human cells. Furthermore, KD of *HINFP* in hRPE cells had multiple deleterious effects including significant loss of cell viability and induction of senescence with upregulated expression of β‐Gal and SASP factors (Figure 3f–h).

We employed serially passaged hRPE cells as an in vitro model to investigate the histone expression changes with aging (Dubey et al., 2023). These experiments showed that RPE cells that reached replicative limits had reduced histone levels, cell proliferation, and upregulation of SASP markers (Figure 4a–g). A study using human diploid fibroblasts (HDFs) as a model for replicative senescence documented a significant reduction of 43%–47% in histones H3 and H4 during late passages, primarily attributed to stress signals associated with telomere shortening. Intriguingly, there was no downregulation of histone H1 in HDFs, indicating that chronic replicative stress selectively affected H3 and H4 histones (O'Sullivan et al., 2010). By contrast, our in vitro data shows ~55% and ~ 85% linker histone H1 loss in late and senescent cells, respectively (Figure 4g). These observations highlighted the specific localization of epigenetic changes to distinct cell types and tissues within an organism.

Given the depletion of histones observed in aged RPE/choroid, we examined the status of histone acetylation, a PTM critical for gene activation and silencing in the cells. Mechanisms of histone acetylation/deacetylation are poorly understood in the RPE in the context of aging. We discovered the loss of global H3 and H4 acetylation in aged RPE coupled with a specific signature of decreased acetyl‐histone marks including H3K14, H3K56, and H4K16 positions (Figure 6a,c–e). This finding aligns with recently reported histone loss and hypoacetylation in aging T‐cells (Kim et al., 2021). Hypoacetylation can lead to chromatin reorganization, and the formation of senescence‐associated heterochromatin foci has been observed in mammalian cells (Contrepois et al., 2012; Zhang et al., 2005). Specific acetylation marks likely have varying roles in regulating aging and lifespan across species and even within different tissues of an organism (Yi & Kim, 2020). For instance, in aged mouse hepatocytes, there is a depletion of H3K14 acetylation, whereas this mark is hyperacetylated in mouse oocytes during both in vivo and in vitro postovulatory aging. Similarly, aged yeast cells were found to have H3K56 hypoacetylation but H4K16 hyperacetylation (Dang et al., 2009). H4K16ac is a critical acetylation mark on H4 that plays a central role in chromatin compaction, gene expression regulation, response to stress, and DNA damage repair. The modulation of H4K16 acetylation significantly impacts age‐related gene expression of the organism, as evidenced in yeast and cellular senescence (Dang et al., 2009; Kozak et al., 2010). Our data provides the first evidence that H4K16 deacetylation is a unique marker to the aging RPE, indicating a potential epigenetic regulatory role in both aging and age‐related retinal diseases. While we did not detect significant loss of HAT and HDAC expression or HDAC activity in aged mouse RPE/choroid, we did observe a downregulatory trend in the expression of HDAC‐2, −6, −8, −10, and Sirt‐1, −7 via RNA‐seq (Figure S4).

In conclusion, this extensive investigation provides an intricate insight into the epigenetic landscape of the aging RPE characterized by significant downregulation of histones, a unique histone expression profile, and a novel signature of histone acetylation. Our in vitro experiments further corroborate these findings, demonstrating that human RPE cells undergoing replicative aging experience reduced levels of histones and enter senescence in later passages. Similarly, aged human eyes exhibit significantly lower histone expression in the RPE layer. Our future studies will investigate the impact of in vitro chronological aging in human RPE on histone expression and cellular senescence. While our investigation primarily focused on the transcriptional regulation of histone biosynthesis through the targeted knockdown of *HINFP*, it is crucial to conduct further research to unravel the intricate molecular cascade involved in modulating histone levels in aging RPE. Histone levels in cells are regulated at multiple levels via transcriptional factors and degradation pathways. The downregulation of transcriptional regulatory factors identified in this study could partially account for the reduced histone levels detected in aged RPE. Thus, further investigation into pathways responsible for histone mRNA and protein degradation is warranted to fully comprehend the underlying mechanisms contributing to this phenomenon. Additionally, the exploration of other histone PTMs, such as methylation, phosphorylation, and ubiquitinoylation, is needed and an area of ongoing investigation. These PTMs are also critical for genomic stability and cell viability and may adversely affect the normal stoichiometry of histones themselves. It is evident from our analyses that the induction of SASP markers, both in vivo and in vitro, is a conserved hallmark of aging in RPE. It is important to acknowledge that our understanding of nucleosome loss during aging and the consequences of histone overexpression on lifespan extension predominantly stems from studies in yeast models highlighting the need for comprehensive studies in mammalian models (Feser et al., 2010; Hu et al., 2014). Recent studies in mammalian models have shown the potential restoration of histone expression by using pharmacological compounds such as rapamycin, metformin, and resveratrol (C. Lin et al., 2020; Lu et al., 2021). These antiaging drugs could potentially target molecules that regulate histone expression and represent an area of future investigation to determine if this approach can reverse histone depletion in the aging RPE. Potential future directions suggested by our findings include investigating alterations in histone variants, histone regulatory factors, and the impact of histone restoration in aging, as this will offer highly innovative insights into aging RPE biology and age‐related diseases. Future research should also address the critical question of whether age‐related histone alterations are a consequence of the aging process itself or actively contribute to aging, pro‐inflammatory gene expression, and cell death. Identifying histone depletion as a hallmark of RPE aging and senescence provides a novel perspective on RPE cell biology, and targeting key regulatory mechanisms of histones holds significant potential for preserving RPE cell viability in numerous age‐related retinal diseases.

## AUTHOR CONTRIBUTIONS

SKD, RD, SCP, and MEK designed research; SKD, RD, SCP, KM, KJ, JR, JA, TW, MG, AGH, and BW performed research; SKD, RD, SCP, KJ, KM, XL, JL, BW, JA, and JR analyzed data; SKD, RD, KJ, SCP, XL, JL, and MEK provided edits of the paper; SKD, RD, and MEK wrote the paper.

## FUNDING INFORMATION

This work was supported by grants from NIH NEI R01EY028206 (Mark E. Kleinman), NIH NEI K08EY021757 (Mark E. Kleinman), BrightFocus Macular Degeneration Research Award (Mark E. Kleinman), American Federation for Aging Research (Mark E. Kleinman), International Retinal Research Foundation (Sushil K. Dubey) and NEI Center Core Grant for Vision Research (NIH NEI P30EY001730; Maureen Neitz).

## CONFLICT OF INTEREST STATEMENT

The authors declare no competing interests.

## Supporting information


Appendix S1

## Data Availability

The data that support the findings of this study are available from the corresponding author upon reasonable request. All RNA‐seq sequencing data have been deposited in the NCBI Gene Expression Omnibus (GEO) under the accession number GSE236221. The subseries of this dataset have been assigned the accession numbers GSE236220 (Total RNA) and GSE236219 (poly(A)‐ enriched).
