# Supplementary material for: Histone deficiency and hypoacetylation in the aging retinal pigment epithelium
Source: Aging Cell. 2024 Feb 26;23(5):e14108. doi: 10.1111/acel.14108 (PMC11113634; doi:10.1111/acel.14108)
Supplement: Supplementary file 1 — Appendix S1 [file ACEL-23-e14108-s001.docx]

**Supporting Information for**

**Histone deficiency and hypoacetylation in the aging retinal pigment epithelium**

*Running title: Histone Profiling in Aging RPE*

Sushil K. Dubey^1*^, Rashmi Dubey^1*^, Subhash C. Prajapati^2^, Kyungsik Jung^1^, Kabhilan Mohan^1^, Xinan Liu^3^, Jacob Roney^4^, Wenjian Tian^1^, Jennifer Abney^4^, Michelle M. Giarmarco^5^, Alvaro G Hernandez^6^, Jinze Liu^7^ and Mark E. Kleinman^1^

^1^Department of Surgery, East Tennessee State University, Johnson City, TN 37614, USA.

^2^Department of Biochemistry and Molecular Genetics, University of Virginia, Charlottesville, VA 22908, USA.

^3^Department of Computer Science, University of Kentucky, Lexington, KY 40506, USA.

^4^Department of Ophthalmology and Visual Sciences, University of Kentucky, Lexington, KY 40536, USA

^5^Department of Ophthalmology, University of Washington, Seattle, WA 98195, USA.

^6^Roy J. Carver Biotechnology Center, University of Illinois at Urbana-Champaign, Urbana, IL 61801, USA.

^7^Department of Biostatistics, Virginia Commonwealth University, Richmond, Virginia 23298-0032, USA.

*SKD and RD contributed equally to this work

**Correspondence:**

Mark E. Kleinman, Department of Surgery, James H. Quillen College of Medicine, East Tennessee State University, Johnson City, TN 37614, USA.

**Email:** [**kleinman@etsu.edu**](mailto:kleinman@etsu.edu)

**Materials and Methods**

**RNA sequencing**

For RNA sequencing analysis, RPE/choroid tissue (2 eyes/sample) was collected from young and aged mice (n=3 per group). RNA extraction was performed using TRIzol reagent (Invitrogen 15596026, Carlsbad, CA) according to the manufacturer's instructions. Subsequently, RNA purification and on-column genomic DNA digestion were conducted using the Pure Link RNA Micro Kit (Invitrogen 12183016, Carlsbad, CA). The quality of RNA samples was assessed using the Agilent 2100 Bioanalyzer system (Agilent, CA), ensuring a median concentration of 25 ng/µl and RIN values >9 for library preparation. Library construction and sequencing on the Illumina HiSeq 2500 platform were carried out at the Roy J. Carver Biotechnology Center, University of Illinois, at Urbana-Champaign. To eliminate host ribosomal RNAs, the Ribo-Zero Gold rRNA Removal Kit (Human/Mouse/Rat) (Illumina MRZG12324, CA) was employed. The rRNA-depleted RNAs were converted into individually barcoded RNAseq libraries with the TruSeq Stranded Total RNA Sample Prep kit (Illumina, CA). To prevent index switching, Unique Dual Indexes (UDIs) were used for barcoding the libraries. Adaptor-ligated double-stranded cDNAs were PCR amplified for 8 cycles using Kapa HiFi polymerase (Kapa Biosystems, MA). The resulting libraries were quantitated using Qubit (ThermoFisher Scientific, MA), and the average cDNA sizes were determined with a Bioanalyzer (Agilent, CA). For accurate pooling of barcoded libraries and maximizing cluster formation in the flowcell, the libraries were diluted to 10nM and further quantitated by qPCR on a CFX Connect Real-Time qPCR system (Biorad, Hercules, CA). The pooled barcoded libraries were multiplexed and loaded onto a single lane for cluster formation and subsequently sequenced on an Illumina HiSeq 2500 platform. Libraries were sequenced from both ends of the fragments, with each end generating 100bp reads. The fastq read files were generated and demultiplexed with the bcl2fastq v 1.8.4 Conversion Software (Illumina, San Diego, CA).

**RNA-seq data mapping and analysis**

Paired-end 100-bp reads were trimmed and filtered using BBDuk (https://github.com/BioInfoTools/BBMap/blob/master/sh/bbduk.sh) and read pairs were mapped to Ensembl GRCm38 transcripts annotation (release 82), using STAR. Transcripts per million (TPM) values were quantified using Sailfish version 0.9.0 with the default parameters using paired-end mode and transformed into normalized counts using the total count method. The main source of variation in the whole transcriptome dataset was determined from the principal component analysis (PCA) of normalized read counts using the “prcomp” function in the R statistical environment (R v. 3.2.3). Differential expression analysis was performed using the Bioconductor’s DESeq2 package and the log2 fold changes, p-values and Benjamin-Hochberg-adjusted p-values (BH) were reported for each gene. A volcano plot of significantly regulated genes was created using the R package EnhancedVolcano. The correlation in gene expression between the rRNA-depleted and poly(A)-selected datasets was calculated using Pearson and visually represented through a correlation plot generated with the R package ggplot2. Genes with adjusted p-value < 0.05 and log2 fold changes greater than or equal to 1 were assigned as differentially expressed genes. ShinyGO (version 0.77) was used for gene ontology enrichment analysis of the top 100 differentially regulated genes (Ge, Jung, & Yao, 2020). Hierarchical clustering and heat maps were generated using the R package heatmap.2 using the log_2_ transformed normalized read counts.

**Histone extraction**

For the preparation of total histones RPE tissues or cellular pellets were resuspended in 1X of diluted Pre-Lysis Buffer (1 ml per 200 mg of tissue and 10^7^ cells/ml) and disaggregated using a hand-held homogenizer. The samples were placed on ice for 10 minutes under stirring to remove the cytosolic fractions. Subsequently, the lysate was centrifuged at 10,000 rpm for 1 min at 4°C, and the supernatant was discarded. The pellet was resuspended in 3 volumes (approximately 200 µl/10^7^ cells or 100 mg of tissue) of Lysis Buffer and incubated on ice for 30 min. Following this, the lysate was centrifuged at 12,000 rpm for 5 minutes at 4 °C to remove the nuclear membranes and the resulting supernatant was transferred to a new tube. For each sample, 0.3 volumes of Balance Buffer were added immediately to the supernatant. The concentration of histones was determined using the Rapid Gold BCA Protein Assay Kit (Pierce A53226, Rockford, IL).

**Western blotting**

Lysates were obtained by treating neural retina, RPE/choroid tissues from mice, and cultured RPE cells with 1X RIPA buffer (Thermo Scientific 89900, Rockford, IL) supplemented with protease (Thermo Scientific, A32963) and phosphatase (Thermo Scientific A32957, Rockford, IL, USA) inhibitor cocktails. The lysates were centrifuged at 12000 rpm for 10 minutes at 4°C, and the protein concentration in the supernatants was quantified using the Rapid Gold BCA Protein Assay Kit (Pierce A53226, Rockford, IL). Equal concentrations of whole cell/tissue lysates (15-25μg) or histone extracts (2-4μg) were resolved on 4–20% or 10-20% Tris-Glycine gels (Thermo Scientific XP04200BOX, Rockford, IL). The proteins were then transferred onto PVDF membranes (Millipore ISEQ00005, Burlington, MA), blocked with 5% skimmed milk in 1X phosphate-buffered saline (PBS) for 1 hour, and incubated with primary antibodies overnight at 4°C on an orbital shaker. Western blot analysis used optimized concentrations of primary antibodies listed in *SI Appendix*, Table S2. Following three washes with PBS-T, the membranes were incubated with HRP-conjugated secondary antibodies (1:5000; Invitrogen G21234, Carlsbad, CA) at room temperature for 2 hours. After three additional washes in PBS-T, the membranes were developed using SuperSignal West Pico PLUS Chemiluminescent Substrate (Thermo Scientific 34580, Rockford, IL) and imaged using a gel imager (Azure Biosystems 300Q, Dublin, CA).

To ensure the uniform loading of samples in Western blot analysis, we utilized GAPDH control and Ponceau-S staining of total cell lysates. This approach was chosen over conventional nuclear protein controls, which are susceptible to variations due to aging or changes in histone levels, as evidenced by prior studies (Angelov et al., 2006; Bin Imtiaz et al., 2021; Gonzalez-Gualda, Baker, Fruk, & Munoz-Espin, 2021; Sen Gupta, Joshi, Pawar, & Sengupta, 2018).

**Immunofluorescence**

Mouse frozen eye sections were processed for immunofluorescence as described previously (Mohan et al., 2023). Following initial fixing and permeabilization, the sections were blocked in a solution containing 4% normal goat serum and 3% bovine serum albumin in 1X PBS for 1h. Sections were incubated with primary antibodies (*SI Appendix*, Table S2) for 1 hour at 37ºC, followed by detection using an appropriate fluorescent-tagged secondary antibody. The nuclei were counter-stained with Hoechst (1:10,000; Invitrogen) for 5 minutes. The following primary antibodies were used for protein detection: Histone H1, H2A, H2B, H3, H4, H3Ac, H4Ac, H4K16Ac, and Isotype (*SI Appendix* Table S2). The images were acquired at 40X magnification using the TCS SP8 confocal microscopy (Leica).

**Immunohistochemistry**

Human paraffin-embedded tissue sections from normal young, adult, and aged donors (kindly provided by Anita Hendrickson, PhD, University of Washington and NDRI) were evaluated by a board-certified ophthalmologist (M.E.K) with H&E staining and light microscopy to confirm a normal appearing posterior segment. Deparaffinization was carried out using xylene, followed by rehydration with decreasing concentrations of ethanol (100% to 95%, 70%, and 50%) for 5 minutes at room temperature for each step. Antigen retrieval was performed using citrate buffer at 114-120°C for 15 minutes. To block endogenous peroxidase activity, the slides were treated with BLOXALL endogenous blocking solution (SP-6000, Vector Lab, CA) for 10 minutes. Subsequently, blocking solution with 2.5% normal goat serum (S-1012-50, Vector Lab, CA) was applied for 30 minutes. The tissue sections were then incubated with primary antibodies targeting Histone H1, H2A, H2B, H3, and H4 (SI Appendix, Table S2) at 4°C overnight. Following this, a biotinylated goat anti-rabbit secondary antibody (BP-2000, Vector Lab, CA) was applied for 1 hour. Immunostaining was visualized by treating the sections with VECTASTAIN ABC-AP (AK-5000, Vector Lab, CA) for 30 minutes and exposing them to an alkaline phosphatase substrate solution (AP-Blue, SK-5300, Vector Lab, CA) until the desired color intensity was achieved. After dehydration, the slides were cover-slipped with VectaMount solution (H-5000, Vector Lab, CA). Imaging and analysis of the stained sections were performed using an Aperio CS2 Scanner (Leica) and Aperio ImageScope software (Leica).

**qPCR**

Total RNA was extracted from hRPE cells and mice neural retina and RPE/choroid tissues as described previously (Mohan et al., 2023) and cDNA was synthesized using the High Capacity cDNA Reverse Transcription Kit (Applied Biosystems 4368814, Lithuania) according to manufacturer’s instructions. The qPCR was performed as described previously (Mohan et al., 2023) using the primer list in Table S3 (*SI* *Appendix* Table S3). Briefly, 20ng cDNA were amplified using the Power SYBR Green PCR Master Mix (Applied Biosystems 4368706, Lithuania) and StepOnePlus Real-Time PCR System (Applied Biosystems, Lithuania). Relative gene expression changes were calculated using either GAPDH as the reference gene. The fold change was calculated by determining the ratio of mRNA levels to control values using the Δ threshold cycle (Ct) method (2^−ΔΔCt^).

**HDAC activity**

To evaluate HDAC activity, RPE/choroid tissues obtained from young and aged mice were lysed using 1X RIPA buffer. Subsequently, tissue lysates (50μg) were analyzed for HDAC activity using the BioVision fluorometric assay kit (Biovision K330, Mountain View, CA), following the manufacturer's instructions.

**Human RPE cell culture and transfection**

Human primary RPE (Lonza, H-RPE 00194987, Walkersville, MD) were cultured in RPE culture medium (RPECM, alpha-MEM plus 1% N1 Supplement, 1% Glutamine-Penicillin-Streptomycin, 1% non-essential amino acids, 250 mg/L taurine, 20 mg/L hydrocortisone, 0.013 mg/L triiodo-thyronine) containing 10% FBS. The cultures were maintained in a humidified atmosphere at 37°C with 5% CO2 and 95% air. Human RPE cells were transfected using HINFP siRNA (ThermoFisher Scientific 4392430, Assay ID s24883, MA) and scrambled-siRNA (ThermoFisher Scientific 4390844, MA) at a final concentration of 75pmol using Lipofectamine 3000 (Invitrogen L3000-015, Carlsbad, CA) as per the manufacturer's instructions.

**Cell proliferation assay**

Cell proliferation assay was performed using 96-well plates. hRPE cells were seeded at a density of 2,500 cells per well. When necessary, cells were transfected with indicated siRNAs. Cell growth was evaluated by MTS assay after culturing cells for a specified duration using The Cell Titer 96® AQueous One Solution Cell Proliferation Assay kit (MTS solution, Promega G3580, Madison, WI) according to the manufacturer’s instruction. Assays were performed in triplicates in three isolates. Results were analyzed by the Unpaired t-test and data were presented as mean± SEM.

***In* *vitro* replicative aging in hRPE cells**

The hRPE cells were maintained in RPECM containing 10% FBS. To induce replicative senescence, early passage (P 3-4) hRPE cells (n = 3 isolates) were grown to 75-80% confluence, serially passaged by repeated trypsinization and subculturing at a 1:4 ratio, until the cells ceased to divide as described before (Matsunaga et al., 1999; Wang, Cui, Nie, Prasad, & Matsubara, 2004). Population doublings (PDLs) for each passage were estimated as two PDLs per passage. Replicative senescence was demonstrated by arrested proliferation, SASP markers, and SA-β-Gal activity increase from PDLs 46-50.

**SA-β-Galactosidase assay**

β-galactosidase activity was measured using Senescence β-Galactosidase Staining Kit (Cell Signaling Technology 9860, MA) according to the manufacturer’s protocol. Briefly, hRPE cells were seeded overnight on 8-chamber glass slides in RPECM media supplemented with 10% FBS. Growth media was removed from individual chambers, and the wells were rinsed with 1X PBS. Cells were fixed with 400µl of 1X fixative solution for 15 minutes, washed twice with PBS and incubated with 400µl of 1X β-galactosidase staining solution for 12-15 hours in a dry incubator at 37°C without CO_2_. After incubation, cells were washed twice with 1X PBS, overlaid with 70% glycerol, and SA-β-Gal positive cells, displaying blue perinuclear staining, were observed under a microscope (Leica, Germany). At least three fields were randomly selected to calculate the positive staining rate.

**Statistical analysis**

For routine statistical analysis, GraphPad Prism (GraphPad Software, La Jolla, USA) was used. The results are presented as mean±standard error of the mean (SEM) or mean±standard deviation (SD). The significance levels were depicted as follows: *p< 0.05; **p< 0.01; ***p< 0.001. For experiments with replicates of paired samples, a two-tailed student’s t-test was applied. Group comparisons were performed using appropriate non-parametric tests, such as Mann-Whitney U or Unpaired t-test.

**Supplementary Figure S1**


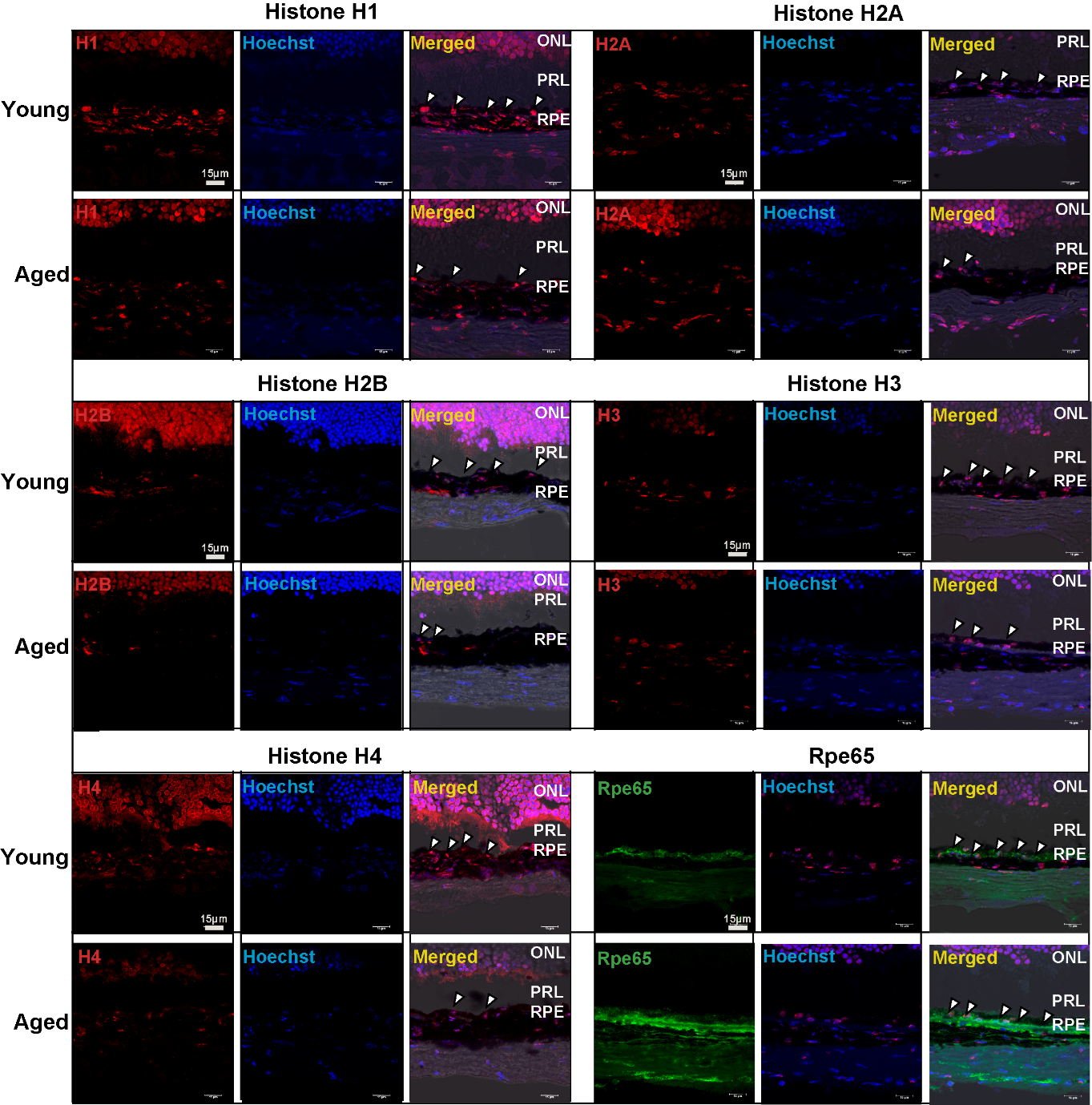


**Supplementary Figure S1:**Representative fluorescence images of histone localization in RPE of young and aged mice. Strong H1, H2A, H2B, H3, and H4 histones (red) expression was found in the RPE (white arrows) of young mice compared to aged mice. Nuclei are stained with Hoechst (blue) in both young and aged retinas. Merged images of red and blue channels with brightfield showed the histone levels in the RPE layer. ONL, outer nuclear layer; PRL, photoreceptor layer, RPE; retinal pigment epithelium. Scale bar: 15μm.

**Supplementary Figure S2**


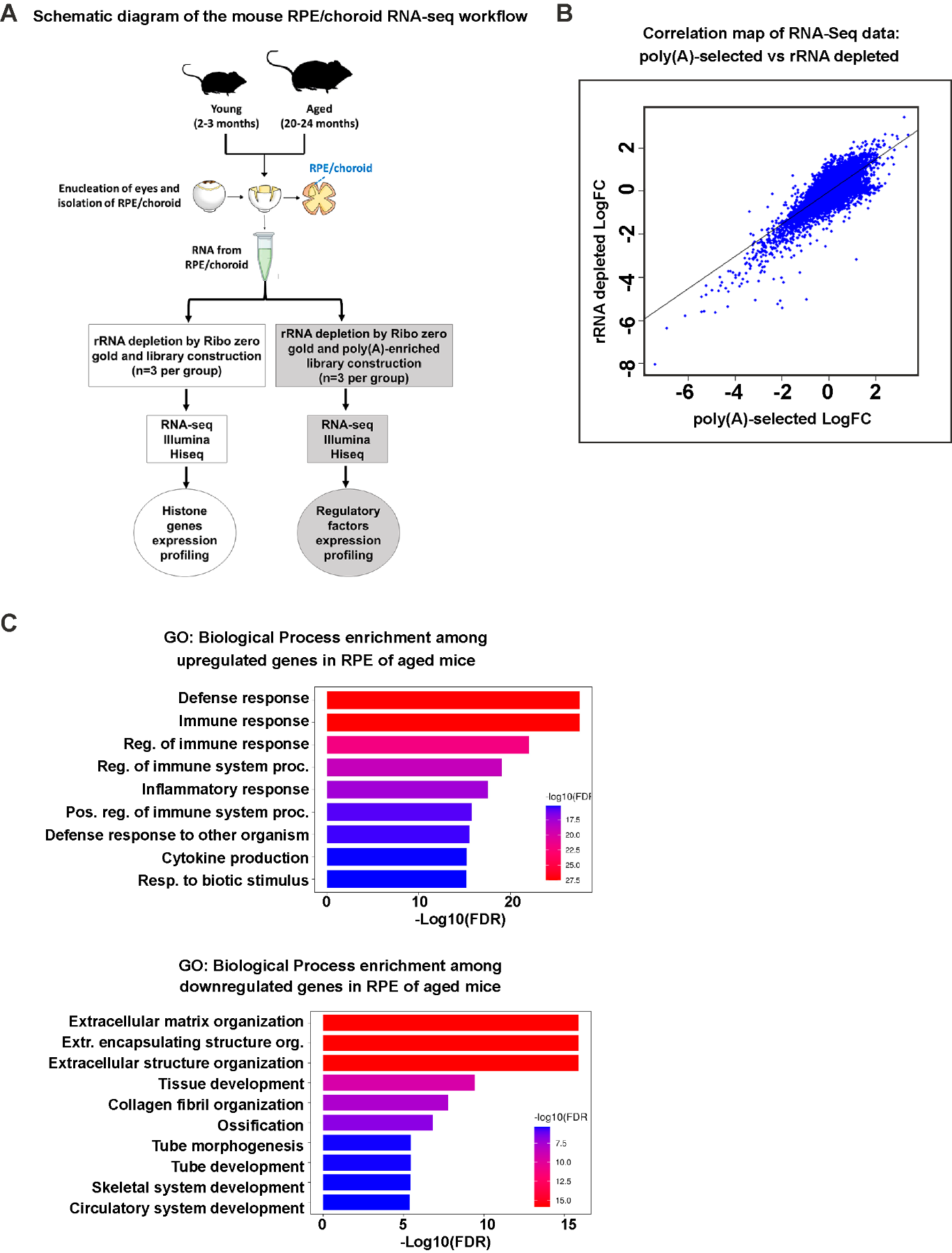


**Supplementary Figure S2:**(A) Schematic representation of the RNA-sequencing experiment conducted on young (n=3, 2-3 months) and aged (n=3, 20-24 months) mice RPE/choroid samples, using ribosomal RNA-depleted and poly(A)-selected techniques. (B) Correlation plot of RNA-Seq data from poly(A) selected and rRNA depleted libraries with linear regression line indicated. (C) The top 10 enriched terms from GO analysis of top 100 upregulated and down-regulated genes from poly(A)-selected RNA-Seq data of young versus aged mice RPE/choroid. The x-axis represents the -log10 scale for the adj. p-value of each GO term.

**Supplementary Figure S3**


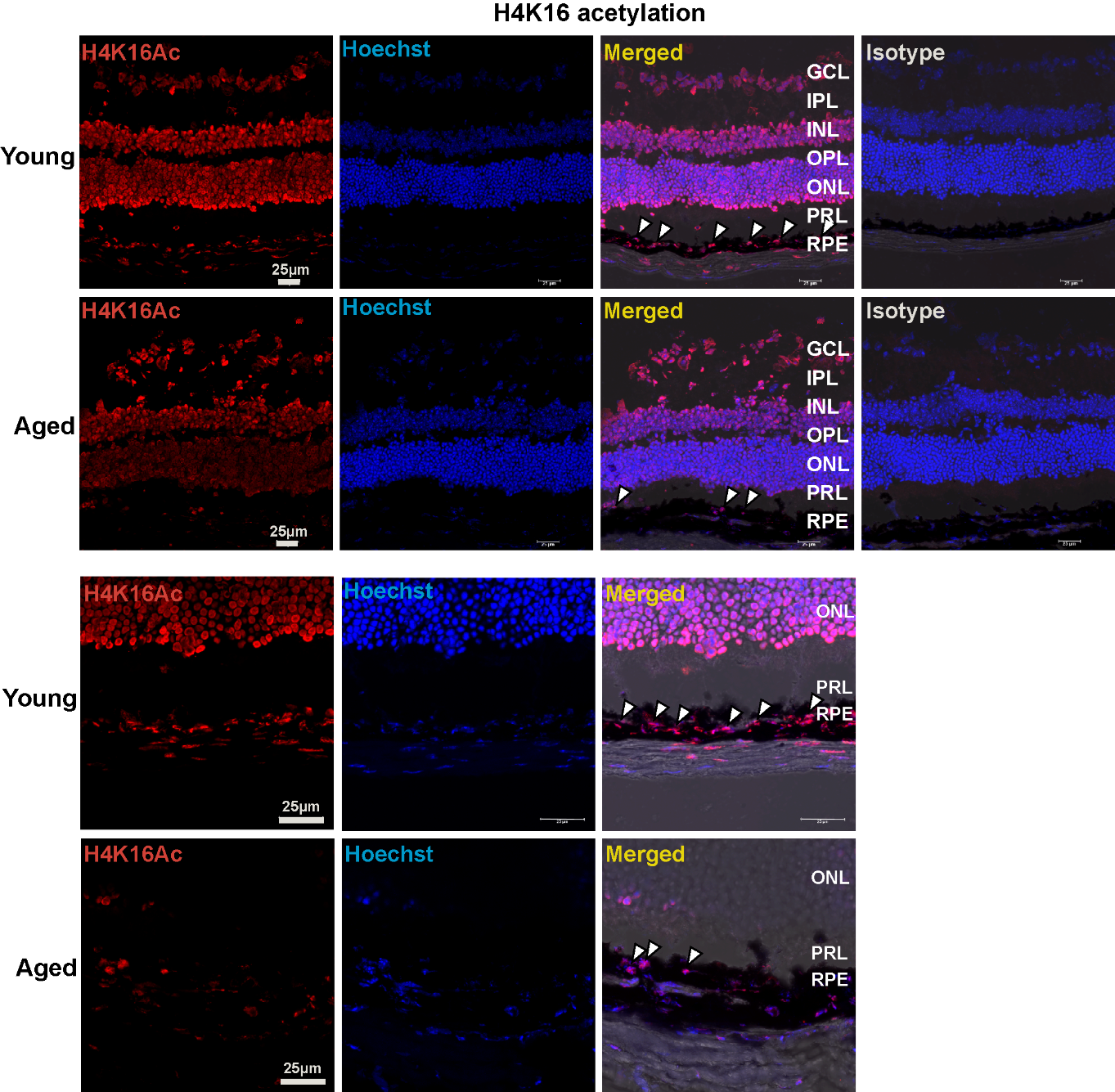


**Supplementary Figure S3:**Representative immunofluorescence images of H4K16 acetylation (red) in RPE of young and aged mice. Nuclei are stained with Hoechst (blue) in both young and aged retinas. Merged images of overlapping red and blue channels with brightfield showed H4K16ac localization in the RPE layer of retinal sections. Scale bar: 25μm.

**Supplementary Figure S4**


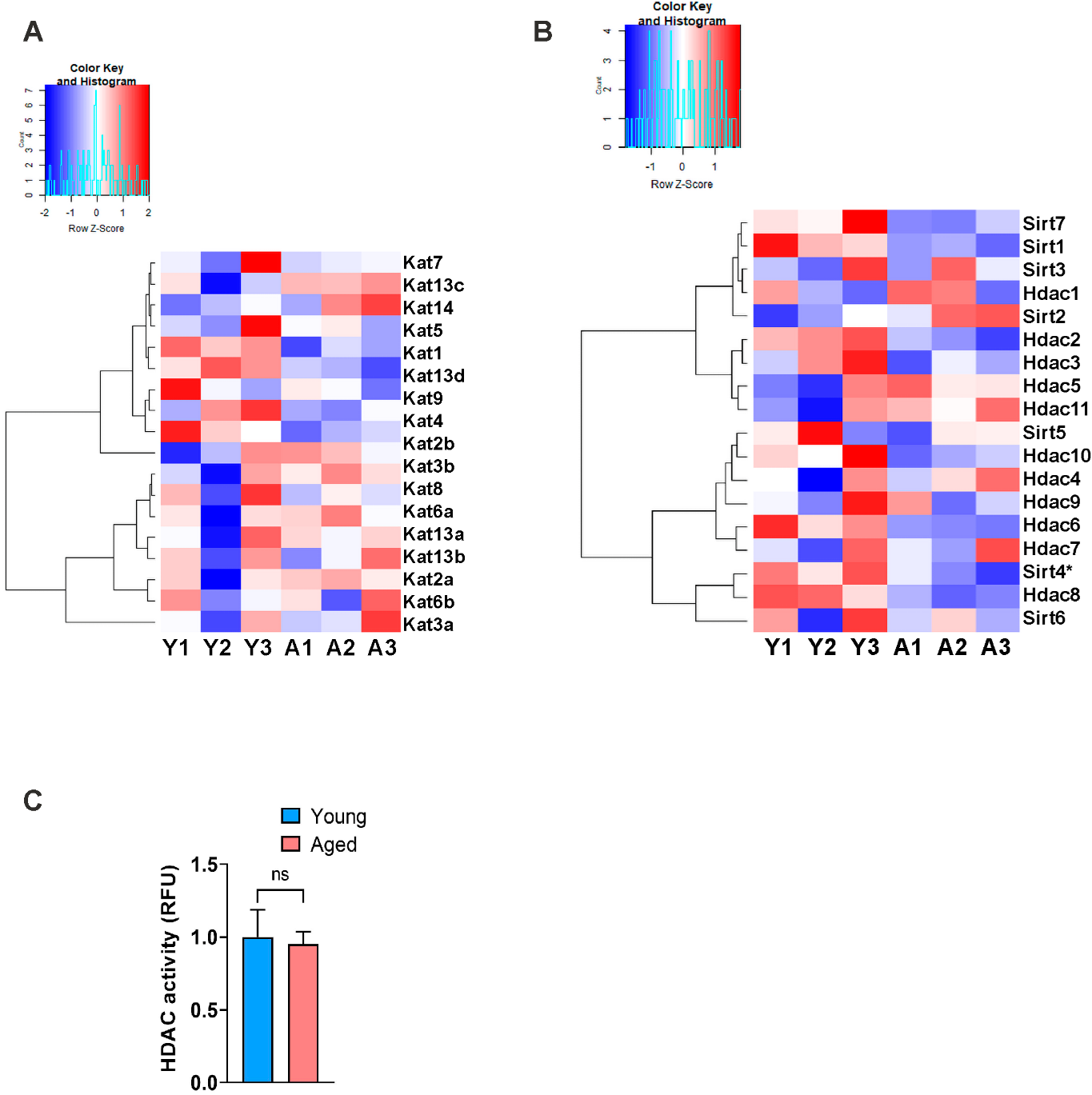


**Supplementary Figure S4:**(A and B) Heatmap representing HAT and HDAC expression in young and aged mice RPE/choroid. * represents adj p-value <0.05. Blue to red represents low to high gene expression. (C) Total HDAC activity was measured in young and aged mice RPE/choroid tissue lysate using a fluorometric assay (n=6 per group). Results were shown as mean±SD.

**Supplemental Table S1**

Expression profile of 114 histone genes in young versus aged RPE/choroid

| **Gene Symbol** | **Gene Name** | **log2 fold change** | **p-adjusted** |
| --- | --- | --- | --- |
| H1f0 | H1.0 linker histone | 0.976 | 5.018E-13 |
| Hist1h2bg | H2B clustered histone 4 | 1.099 | 5.300E-06 |
| Hist1h1e | H1.4 linker histone, cluster member | 0.806 | 6.379E-05 |
| Hist2h2be | H2B clustered histone 21 | 0.808 | 1.442E-04 |
| Hist1h3d | H3 clustered histone 1 | 1.243 | 1.866E-04 |
| Hist1h1d | H1.3 linker histone, cluster member | 0.731 | 4.808E-04 |
| Hist2h2bb | H2B clustered histone 18 | 0.761 | 6.375E-04 |
| Hist1h4m | H4 clustered histone 1 | 1.072 | 1.408E-03 |
| Hist4h4 | H4 clustered histone 1 | 0.906 | 1.317E-02 |
| Hist1h3b | H3 clustered histone 1 | 0.932 | 1.384E-02 |
| H2afy2 | macroH2A.2 histone | 0.574 | 1.618E-02 |
| Hist1h2bf | H2B clustered histone 4 | 1.078 | 1.730E-02 |
| Hist1h2bn | H2B clustered histone 3 | 0.725 | 2.427E-02 |
| Hist1h2bk | H2B clustered histone 12 | 0.855 | 2.565E-02 |
| Hist1h2ae | H2A clustered histone 4 | 0.740 | 2.726E-02 |
| Hist1h2af | H2A clustered histone 4 | 0.945 | 2.879E-02 |
| Hist1h3e | H3 clustered histone 6 | 0.560 | 3.684E-02 |
| Hist1h3i | H3 clustered histone 1 | 0.864 | 3.792E-02 |
| Hist1h2bc | H2B clustered histone 4 | -0.430 | 3.979E-02 |
| Hist1h3c | H3 clustered histone 1 | 0.812 | 4.358E-02 |
| Hist2h3b | H3 clustered histone 13 | 0.763 | 0.052 |
| Hist1h2ab | H2A clustered histone 4 | 0.691 | 0.058 |
| Hist1h2bm | H2B clustered histone 3 | 0.782 | 0.060 |
| Hist1h2ak | H2A clustered histone 4 | 0.664 | 0.067 |
| Hist1h3h | H3 clustered histone 1 | 0.679 | 0.094 |
| Hist1h2bl | H2B clustered histone 7 | 0.876 | 0.102 |
| Hist1h2bb | H2B clustered histone 3 | 0.430 | 0.109 |
| H3f3a | H3.3 histone A | 0.289 | 0.118 |
| Hist2h4 | H4 clustered histone 1 | 0.553 | 0.141 |
| Hist1h2an | H2A clustered histone 4 | 0.802 | 0.144 |
| Hist1h1b | H1.5 linker histone, cluster member | 0.484 | 0.149 |
| Hist1h4d | H4 clustered histone 4 | 0.474 | 0.156 |
| Hist1h2ai | H2A clustered histone 4 | 0.516 | 0.172 |
| Hist1h2bj | H2B clustered histone 11 | 0.516 | 0.176 |
| Hist1h4a | H4 clustered histone 1 | 0.512 | 0.185 |
| Hist1h4b | H4 clustered histone 1 | 0.573 | 0.188 |
| Hist1h3a | H3 clustered histone 1 | 0.608 | 0.194 |
| Hist2h3c1 | H3 clustered histone 14 | 0.486 | 0.206 |
| H2afj | H2J.A histone | -0.317 | 0.206 |
| Hist2h2ac | H2A clustered histone 20 | 0.397 | 0.217 |
| Hist1h2ah | H2A clustered histone 12 | 0.785 | 0.220 |
| Hist1h2ad | H2A clustered histone 4 | 0.600 | 0.224 |
| Hist2h2aa2 | H2A clustered histone 18 | 0.617 | 0.261 |
| Hist1h2ag | H2A clustered histone 11 | 0.620 | 0.274 |
| Hist3h2a | H2A clustered histone 25 | -0.219 | 0.276 |
| Hist1h4j | H4 clustered histone 6 | 0.445 | 0.289 |
| Hist1h2bh | H2B clustered histone 4 | 0.464 | 0.355 |
| Gm6421 | predicted pseudogene 6421 | 0.206 | 0.399 |
| Hist1h3g | H3 clustered histone 1 | 0.509 | 0.428 |
| Hist1h1a | H1.1 linker histone, cluster member | 0.272 | 0.446 |
| Hist1h2be | H2B clustered histone 4 | 0.194 | 0.484 |
| Hist1h3f | H3 clustered histone 7 | 0.261 | 0.499 |
| Hist1h4f | H4 clustered histone 3 | 0.291 | 0.508 |
| Hist1h4h | H4 clustered histone 8 | 0.201 | 0.575 |
| Hist1h4k | H4 clustered histone 11 | 0.214 | 0.657 |
| Gm10257 | predicted gene 10257 | -0.196 | 0.687 |
| Hist3h2bb-ps | H2B clustered histone 26 | 0.196 | 0.729 |
| Hist1h1c | H1.2 linker histone, cluster member | 0.056 | 0.803 |
| Hist1h4c | H4 clustered histone 3 | 0.129 | 0.860 |
| Hist1h2ac | H2A clustered histone 4 | -0.064 | 0.867 |
| Cenpa | centromere protein A | -0.093 | 0.870 |
| Hist1h2bp | H2B clustered histone 22 | 0.131 | 0.881 |
| H1fx | H1.10 linker histone | 0.125 | 0.882 |
| Hist2h3c2 | H3 clustered histone 2 | -0.048 | 0.935 |
| Hist1h4n | H4 clustered histone 18 | 0.041 | 0.951 |
| H2afy | macroH2A.1 histone | 0.016 | 0.952 |
| Hist1h4i | H4 clustered histone 8 | 0.022 | 0.956 |
| H3f3c | predicted gene 7426 | 0.018 | 0.958 |
| H2afv | H2A.Z histone variant 2 | -0.015 | 0.966 |
| H2afx | dolichyl-phosphate N-acetylglucosaminephosphotransferase 1 | -0.017 | 0.973 |
| Hist3h2ba | H2B clustered histone 27 | -0.012 | 0.987 |
| H2afz | H2A.Z variant histone 1 | -0.006 | 0.987 |
| 1700012L04Rik | H2A histone family member L1M | 0.016 | NA |
| 1700014N06Rik | H2B.W histone 2 | NA | NA |
| 1700024P04Rik | H2B.L histone variant 1 | NA | NA |
| 1700054O13Rik | H2A.P histone | 0.478 | NA |
| Gm11336 | H2A clustered histone 5, pseudogene | 0.187 | NA |
| Gm12260 | H3.4 histone, cluster member | -0.612 | NA |
| Gm14474 | H2A histone family member L1B | NA | NA |
| Gm14475 | H2A histone family member L1I | 0.116 | NA |
| Gm14476 | H2A histone family member L1D | NA | NA |
| Gm14477 | H2A histone family member L1C | NA | NA |
| Gm14478 | H2A histone family member L1H | NA | NA |
| Gm14479 | H2A histone family member L1F | NA | NA |
| Gm14482 | H2A histone family member L1G | NA | NA |
| Gm14483 | H2A histone family member L1A | NA | NA |
| Gm14484 | H2A histone family member L1E | NA | NA |
| Gm14501 | H2A histone family member L1K | -0.102 | NA |
| Gm14920 | H2A.B variant histone 1 | NA | NA |
| Gm16501 | H2A histone family member L2B | NA | NA |
| Gm4906 | H2A histone family member L1J | 0.138 | NA |
| Gm5132 | H2A histone family member L1O | 0.116 | NA |
| Gm5382 | H2A histone family member L1N | NA | NA |
| Gm6026 | H2A histone family member L2C | NA | NA |
| Gm6970 | H1.11 linker histone, pseudogene | -0.220 | NA |
| H1fnt | H1.7 linker histone | 0.140 | NA |
| H1foo | H1.8 linker histone | 0.309 | NA |
| H2afb1 | H2A.B variant histone 1 | 0.143 | NA |
| H2afb2-ps | H2A.B variant histone 2 | NA | NA |
| H2afb3-ps | H2A.B variant histone 3 | NA | NA |
| H2afy3 | macroH2A.3 histone | 0.069 | NA |
| H3f3b | H3.3 histone A | 0.125 | NA |
| Hils1 | H1.9 linker histone | 0.117 | NA |
| Hist1h1t | H1.6 linker histone, cluster member | -0.081 | NA |
| Hist1h2aa | H2A clustered histone 1 | -0.048 | NA |
| Hist1h2aj | H2A clustered histone 10 | NA | NA |
| Hist1h2al | H2A clustered histone 8 | -0.342 | NA |
| Hist1h2ao | H2A clustered histone 23 | -0.102 | NA |
| Hist1h2ap | H2A clustered histone 24 | 0.116 | NA |
| Hist1h2ba | H2B clustered histone 1 | 0.035 | NA |
| Hist1h2bq | H2B clustered histone 4 | -0.101 | NA |
| Hist1h2br | H2B clustered histone 24 | 0.116 | NA |
| Hist2h2aa1 | H2A clustered histone 18 | NA | NA |
| Hist2h2ab | H2A clustered histone 21 | NA | NA |

Cells highlighted in yellow are significantly altered between young and aged RPE/choroid.

**Supplementary Table S2**

| **Antibody** | **Company** | **Catalog No.** | **Dilutions** |
| --- | --- | --- | --- |
| Histone H1 | ThermoFisher Scientific | PA5-30055 | 1:2000 (WB), 2 μg/ml (IF), 5 μg/ml (IHC) |
| H2A | Abcam | ab18255 | 1:2000 (WB), 2 μg/ml (IF) |
| H2B | Abcam | ab1790 | 1:5000 (WB) |
| H2B | GeneTex | GTX115955 | 5 μg/ml (IF), 5 μg/ml (IHC) |
| H3 | Abcam | ab1791 | 1:25000 (WB), 5 μg/ml (IHC) |
| H3 | GeneTex | GTX122148 | 1 μg/ml (IF) |
| H4 | Novus Biologicals | NBP2-42856 | 1:2000 (WB) |
| H4 | Proteintech | 16047-1-AP | 1 μg/ml (IF) |
| H4 | Abcam | Ab10158 | 5 μg/ml (IHC) |
| Pan-H3ac | Abcam | ab47915 | 1:3000 (WB) |
| Pan-H3ac | Active Motif | 61637 | 1 μg/ml (IF) |
| H3K9ac | Millipore Sigma | 06-942 | 1:1000 (WB) |
| H3K14ac | Millipore Sigma | 04-1044 | 1:500 (WB) |
| H3K27ac | Millipore Sigma | 07-360 | 1:5000 (WB) |
| H3K56ac | Abcam | ab76307 | 1:2000 (WB) |
| Pan-H4ac | Abcam | ab177790 | 1:20000 (WB), 2 μg/ml (IF) |
| H4K5ac | Abcam | ab51997 | 1:10000 (WB) |
| H4K8ac | Abcam | ab45166 | 1:5000 (WB) |
| H4K12ac | Abcam | an177793 | 1:2500 (WB) |
| H4K16ac | Abcam | ab109463 | 1:2000 (WB), 2 μg/ml (IF) |
| H4K16ac | Active Motif | 39929 | 2 μg/ml (IF) |
| GAPDH | Santa Cruz Biotechnology | sc-25778 | 1:3000 (WB) |
| HINFP | Proteintech | 10066-2-AP | 1:800 (WB) |

List of primary antibodies used in this study.

WB: Western blot, IF: Immunofluorescence, IHC: Immunohistochemistry

**Supplementary Table S3**

List of qPCR primers used in this study

| **Primer Name** | **Forward** | **Reverse** |
| --- | --- | --- |
| **Mouse** |  |  |
| mHist1h1d | AAGCCTAAGAAGGCGACTGG | CTTGGCTGGACTCTTTGCTG |
| mHist1h1e | CTCTCTCCTCACACGCTTCG | GCCTTGGTGATGAGTTCGGA |
| mH1F0 | GATGAGCCCAAAAGGTCGGT | CTTCTTGACAGGGGTGGCTT |
| mPanH2b | TACAACAAGCGCTCGACCAT | AGCTGGTGTACTTGGTGACG |
| mHist1H3b | CTGATCCGCAAGCTGCCGTTC | GTTGGTGTCCTCAAACAGACCC |
| mHist1H3i | TTCTAGTGTACTGAGATGGCTCGT | GTAGCGGTGAGGCTTCTTCA |
| mHist2h4 | TAGCCATGTCTGGCAGAGG | CTGGATGTTGTCACGCAAGA |
| mHist1H4m | TGCTTCGTCTTAGAGCAGTACAGT | CGTGATGCCCTGGATGTTAT |
| mCasp8ap2 | GCCCTCTGAATAGTCCAGTGAG | ATCAGACTGACTCTTGAAGGTAGA |
| mNpat | CAGTGCGTTTGCTGTCAGCCAA | CCACCATTCCTTGAAGTACAGGC |
| mHinfp | CATGCGCTTTCGACACAGTGAG | CACACCTGTAGGCTGACTCCTT |
| mPdhb | CATCTCGTGACTGTGGAAGGAG | ACATCAGCACCAGTGACACGCA |
| **Human** |  |  |
| hHINFP | GCAACCACATGCGCTTTCGTCA | CTGTGGGTATCCAGGTGCTTCT |
| hHIST1H1B | CTAAGGAGCGCAATGGCCTTT | CTTCGGAGTCTTCTTCACTGC |
| hHIST2H2AB | CCATCTGCAACTAGCCGTGAG | CAGGCTTGTGACTCTCCGT |
| hHIST1H2AE | CTACTCCGAACGAGTCGGG | GATGGTCACGCGACCTAGAAG |
| hHIST1H2BF | ACCTGCTAAGTCCGCTCCT | CTACGCTTGCGCTTCTTACCA |
| hHIST1H3A | ACTGCTCGGAAGTCTACTGGT | GCGCTGGAAAGGTAGTTTACGA |
| hHIST1H3B | ATGGCTCGTACTAAACAGACAGC | TTCCGAATCAGCAACTCGGTC |
| hHIST1H3D | CCATTCCAGCGTCTAGTCCG | TCTGAAAACGCAGATCAGTCTTG |
| hH4A,N,O | GAGACAACATTCAGGGCATCAC | GAGGCCAGAGATCCGCTTAA |
| hH4C | AAGTTAAGAGTTGTTGTTTGTCTTCG | CCACCTTTGCCTCTACCAGA |
| hH4D,E | TGGGTGAGACTCCTCTTGCT | AAGACCCTTCCCGCCTTT |
| hCCL5 | CCTGCTGCTTTGCCTACATTGC | ACACACTTGGCGGTTCTTTCGG |
| hCXCL8 | GAGAGTGATTGAGAGTGGACCAC | CACAACCCTCTGCACCCAGTTT |
| hIL6 | ATCTGGATTCAATGAGGAGACTTG | GGAACTGGATCAGGACTTTTGTACT |
| hMMP3 | CACTCACAGACCTGACTCGGTT | AAGCAGGATCACAGTTGGCTGG |
| hMMP12 | GATGCTGTCACTACCGTGGGAA | CAATGCCAGATGGCAAGGTTGG |
| hIL1B | CCACAGACCTTCCAGGAGAATG | GTGCAGTTCAGTGATCGTACAGG |
| hICAM1 | AGCGGCTGACGTGTGCAGTAAT | TCTGAGACCTCTGGCTTCGTCA |

Angelov, D., Bondarenko, V. A., Almagro, S., Menoni, H., Mongelard, F., Hans, F., . . . Bouvet, P. (2006). Nucleolin is a histone chaperone with FACT-like activity and assists remodeling of nucleosomes. *EMBO J, 25*(8), 1669-1679. doi:10.1038/sj.emboj.7601046

Bin Imtiaz, M. K., Jaeger, B. N., Bottes, S., Machado, R. A. C., Vidmar, M., Moore, D. L., & Jessberger, S. (2021). Declining lamin B1 expression mediates age-dependent decreases of hippocampal stem cell activity. *Cell Stem Cell, 28*(5), 967-977 e968. doi:10.1016/j.stem.2021.01.015

Ge, S. X., Jung, D., & Yao, R. (2020). ShinyGO: a graphical gene-set enrichment tool for animals and plants. *Bioinformatics, 36*(8), 2628-2629. doi:10.1093/bioinformatics/btz931

Gonzalez-Gualda, E., Baker, A. G., Fruk, L., & Munoz-Espin, D. (2021). A guide to assessing cellular senescence in vitro and in vivo. *FEBS J, 288*(1), 56-80. doi:10.1111/febs.15570

Matsunaga, H., Handa, J. T., Aotaki-Keen, A., Sherwood, S. W., West, M. D., & Hjelmeland, L. M. (1999). Beta-galactosidase histochemistry and telomere loss in senescent retinal pigment epithelial cells. *Invest Ophthalmol Vis Sci, 40*(1), 197-202.

Mohan, K., Dubey, S. K., Jung, K., Dubey, R., Wang, Q. J., Prajapati, S., . . . Kleinman, M. E. (2023). Long-Term Evaluation of Retinal Morphology and Function in Rosa26-Cas9 Knock-In Mice. *Int J Mol Sci, 24*(6). doi:10.3390/ijms24065186

Sen Gupta, A., Joshi, G., Pawar, S., & Sengupta, K. (2018). Nucleolin modulates compartmentalization and dynamics of histone 2B-ECFP in the nucleolus. *Nucleus, 9*(1), 350-367. doi:10.1080/19491034.2018.1471936

Wang, X. F., Cui, J. Z., Nie, W., Prasad, S. S., & Matsubara, J. A. (2004). Differential gene expression of early and late passage retinal pigment epithelial cells. *Exp Eye Res, 79*(2), 209-221. doi:10.1016/j.exer.2004.03.013
